# Global patterns of natural selection inferred using ancient DNA

**DOI:** 10.64898/2026.01.07.697984

**Published:** 2026-01-08

**Authors:** Laura L. Colbran, Jonathan Terhorst, Iain Mathieson

**Affiliations:** 1 Department of Genetics, Perelman School of Medicine, University of Pennsylvania, Philadelphia, PA, 19104, USA.; 2 Department of Statistics, University of Michigan, Ann Arbor, MI, 48109, USA.

## Abstract

Ancient DNA has revolutionized our understanding of human history, and is now yielding important insights into evolution and natural selection. However, studies of selection using ancient DNA have largely been limited to Europe, excluding populations in other parts of the world. While many selective pressures were local to specific populations others, for example those related to the development of agriculture, may have been universal. By studying a broader range of global populations, we can identify examples of local adaption but also more general principles of adaptation to climatic, social and technological changes.

We therefore leverage ancient DNA to test for selection in 7244 individuals from 13 ancient and 19 present-day populations across five regions: Europe, East Asia, South Asia, Africa and the Americas. In each region, we tested for selection using multiple approaches that account for complex demographic histories. We identify 31 genome-wide significant signals of selection, including both known and novel loci. We find a high degree of shared signal across regions, suggesting extensive parallel or shared adaptation. Using a novel admixture-aware time series method, we find that the strength of selection on many variants changed over time, for example decreasing selection at *LCT* in Europe and increasing selection at *ADH1B* in East Asia over the past few thousand years.

Finally, we developed a test for polygenic selection on complex traits by modeling the frequencies of trait-associated alleles identified in GWAS. We tested for selection jointly across regions, avoiding the confounding effect of population stratification by excluding the European or East Asian GWAS population from the selection test. We find evidence for directional selection on pigmentation and immune traits, and that strong stabilizing selection on female waist-hip ratio was universal across human populations suggesting a fundamental constraint on human morphology.

## Introduction

As modern humans migrated out of Africa and throughout the world over the last 50,000 years, they encountered dramatically different environmental pressures, providing the opportunity for genetic adaptation to shape human variation. In the past 10,000 years (the Holocene), populations in different parts of the world independently developed agriculture, urbanism and new forms of social organization. This raises the question of how humans adapted to these new environments, how much those adaptations were shared across populations, and how they affect human diversity today. Understanding which parts of the genome have been subject to selection can highlight functionally important regions, and contribute to understanding how they influence phenotype. Further, strongly selected regions can play a role in medically-significant phenotypes, regardless of their current fitness cost, making patterns of selection informative for understanding variation in health outcomes among populations.

The emerging science of ancient DNA has brought a new perspective to studies of human evolution. The ability to observe allele frequency changes directly combined with a better understanding of demographic history has enabled powerful inference of natural selection [1], precise estimates of timing [2, 3], and the ability to recover signatures of selection that have been obscured by admixture or genetic drift [4, 5]. However, due to sampling limitations, large-scale ancient DNA selection studies have been limited to Europe [1, 6, 7, 3, 8], limiting the scope for comparative evolutionary studies.

We therefore took advantage of the recent influx of non-European ancient DNA to conduct a scan for selection across five regions of the world—Africa, East Asia, Europe, South Asia, and the Americas—each with hundreds of ancient samples and present-day population data. Each of these regions has a complex history of Holocene admixture and we adapted an allele frequency-based test [1] to account for more general admixture history. We further adapted this test to detect polygenic selection, by modeling expected frequencies of alleles associated with a complex trait. One of the primary advantages of ancient DNA is that ancient individuals can be directly dated so we also developed a time series approach to model the evolution of allele frequencies in admixed populations. We find evidence of both private and shared targets of selection, demonstrating the power of ancient DNA to highlight the interconnected nature of evolution in the recent human past.

## Results

### An allele frequency-based test for selection

**Figure 1:**
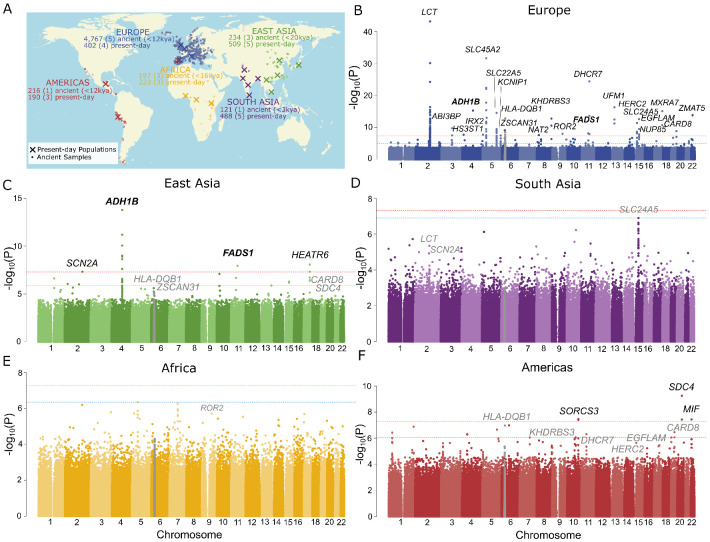
Scan for selection on 1240k variants based on admixture proportions for 5 regions highlighted in A). Manhattan plots of the results for B) Europe, C) East Asia, D) South Asia, E) Africa, and F) the Americas. Significant SNPs are filtered to only include those with at least one more nearby (at least 2 more for Europe), in high LD, and also passing FDR<0.1 (Methods). The HLA is indicated in grey, as it was treated independently of the rest of the genome. Peaks are labeled with the causal gene if known, or the nearest to the lead SNP. Bold text indicates loci passing genome-wide multiple testing in multiple regions, while grey text labels loci that are genome-wide significant in another region, and nominally significant in the indicated region after correcting for the total number of genome-wide significant peaks.

Identifying natural selection in genomic data requires distinguishing its effects from the effects of admixture and genetic drift. The Holocene is characterized by high levels of admixture between previously diverged populations, which can lead to both false positives and false negatives in selection scans [4, 5, 8]. We therefore developed a test where we model expected allele frequency based on genome-wide admixture proportions, extending a previous approach [1] by modeling the ancient populations themselves as admixtures of unobserved source populations. This approach is similar to that of Cheng et al. [9], except that we treat source populations as independent. We perform a likelihood ratio test and then apply a correction for the inflation in test statistics due to genetic drift by calculating P-values from a gamma distribution fitted to the distribution of test statistics. We applied this method to test for selection at 1,150,640 variants captured by the 1240k reagent [10, 11] for five regions with a genetic record which includes both ancient and present-day samples: Africa, East Asia, Europe, South Asia, and Central/South America (Fig. 1A). In each region, we defined populations of ancient and present-day individuals based on genome-wide ancestry and inferred ancestry proportions from the unobserved source populations using ADMIXTURE [12] (Supplementary Figures 1-5).

### 31 signals of selection with extensive sharing across continents

After filtering out SNPs with potential genotype errors, we identified 24, 4, and 3 genome-wide significant (*p* < 5 × 10^−8^) peaks in Europe, East Asia, and the Americas respectively, with none identified in South Asia or Africa (Fig. 1B-F, Supplementary Table 1, Supp. Fig. S1). The differing numbers of peaks reflect the relative power we have in each region; simulations show that we have the most power to detect selection in Europe–though still only around 50% for a selection coefficient of 0.02—and the least in Africa (Supplementary Note A). Comparison with other selection scans conducted using European data suggests that our ordering of loci is similar, and the main factor affecting the number of loci reported is the difference in the genome-wide significance threshold (Supplementary Note B).

The loci we detect include well-known signals of selection associated with diet, pigmentation and the immune system, as well as novel loci of unknown function (Supplementary Table 1, Supplementary Note C). Two loci were genome-wide significant in more than one region (*ADH1B* and *FADS1* in both Europe and East Asia), and twelve were genome-wide significant in one region and nominally significant (at P<0.05/31) in at least one other. Variants passing FDR< 0.05 (N=215) in Europe, where we had the highest power, are significantly enriched in the lower tail of the p-value distribution for all other non-African regions (P = 9.0 × 10^−8^, 1.5 × 10^−5^, 9.2 × 10^−11^ and 0.45 for East Asia, the Americas, South Asia, and Africa, respectively; Kolmogorov-Smirnov test). Overall, this suggests that a relatively high proportion of selected variants have shared signals across regions, reflecting either parallel selection on standing variation, selection on recurrent mutations at the same loci, or selection prior to the most recent shared ancestry.

### Admixture-aware modeling of time series data

**Figure 2:**
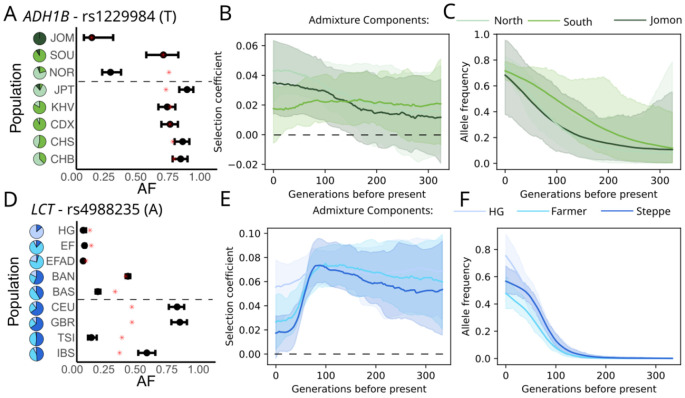
Ancestry-specific time series of the most strongly selected loci in East Asia and Europe. A) Observed allele frequencies; B) inferred selection coefficients; C) Allele frequency trajectories in admixture components at *ADH1B* in East Asia and D-F) *LCT* in Europe. We plotted the information for the derived allele of the lead SNP in each peak. In the frequency plots (first column), the bars represent 95% confidence intervals, while the red asterisk indicates the fitted frequency from the admixture model and the dashed line separates ancient (top) from present-day (bottom) groups. The pie charts indicate the average proportion of each admixture component for each population. See Methods for population abbreviations. In the plots of inferred selection coefficient and allele frequency (second and third columns), different colors distinguish the k source populations, solid lines represent fitted values, and shaded areas represent the 5-95% bootstrap confidence interval.

We extended our previous time-series modeling approach [bmws; 2] to take into account varying levels of admixture among individuals. We model allele frequencies evolving separately in each admixture component, allowing selection coefficients to vary over time and across admixture components in a regularized manner. We conducted simulations to validate the approach (Supplementary Text) then applied it to all significant loci. This allows us to identify changes in the strength of selection over time. For example, at *ADH1B*—an alcohol dehydrogenase gene where a variant associated with poor alcohol metabolism is known to have been under strong selection in East Asia [13-16]—we find a distinct increase in the selection coefficient between 100 and 150 generations ago (Fig. 2A-C). The *ADH1B* locus also shows evidence of selection in Europe (Supp. Fig. S2), also previously identified [8, 17]. However, the putatively causal East Asian variant (rs1229984) is rare in Europe and it remains unclear whether the phenotype under selection is shared.

At *LCT*, the strongly selected variant associated with adult lactase persistence in Europe [18], we infer strong selection up to about 100 generations ago, consistent with previous estimates of timing [2, 3] (Fig. 2D-F). Selection subsequently drops off into the present-day, consistent with several lines of evidence showing little or no recent selection [19] ,or phenotypic effects today [20, 21].

### Parallel adaptation of fatty acid metabolism in Europe and East Asia

**Figure 3:**
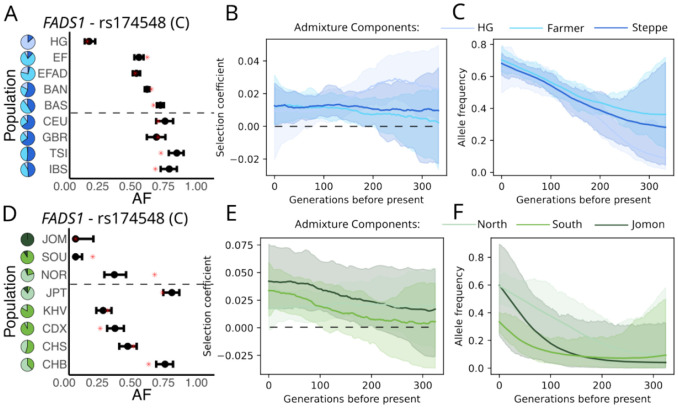
Details of the lead SNP at the *FADS1* locus in Europe and East Asia. A) Plot of observed Allele Frequencies, B) Inferred Selection coefficients, and C) AF trajectories in admixture components at *FADS1* in Europe, and in D-F) East Asia. We plotted the derived allele of rs174548, which is the lead SNP in Europe. Details of the plots are the same as in Fig. 2.

Selection for the same *FADS1* haplotype has been previously identified in both Europe [1] and East Asia [22, 23]. The selected haplotype increases the rate of synthesis of plasma unsaturated fatty acids, is believed to be an adaptation to a relatively plant-heavy agricultural diet [24], and appears to still be under selection today in Europe [21]. We estimate that selection in Europe remained relatively constant through time with a selection coefficent around 1% (Fig. 3A-C). In contrast, in East Asia, we find that selection has intensified over time, with the allele frequency increasing substantially in the past 100 generations (Fig. 3D-F).

### Selection on pigmentation in Europe

**Figure 4:**
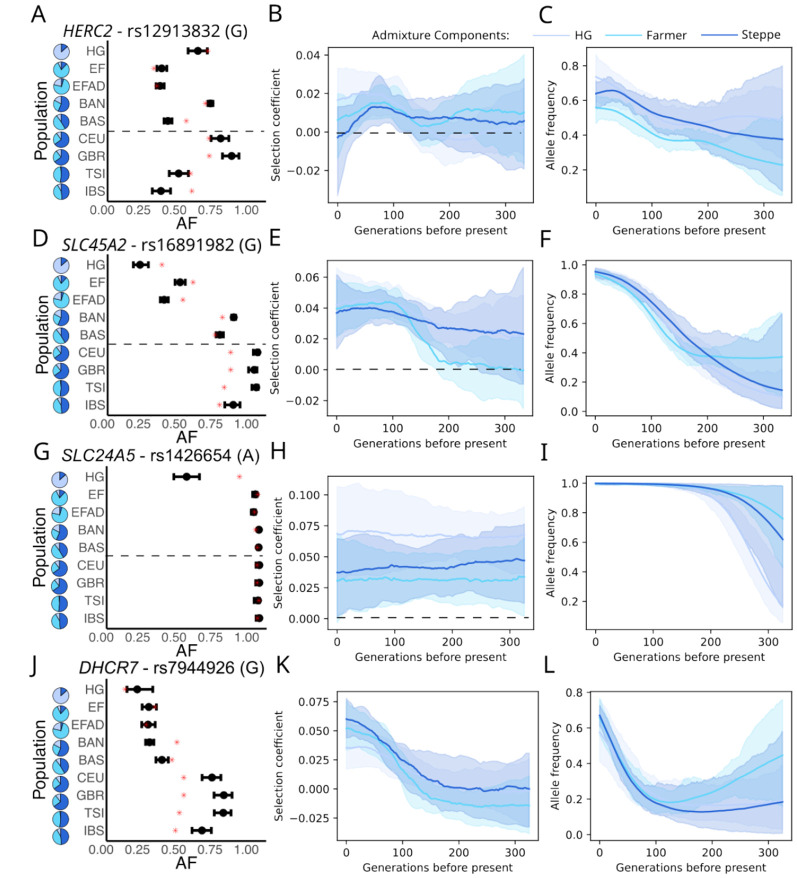
Details of the *HERC2, SLC45A2* and *SLC24A5* loci in Europe. A) Plot of observed Allele Frequencies, B) Inferred Selection coefficients, and C) AF trajectories in admixture components at *Herc2*, followed by D-F) *SLC45A2*, G-I) *SLC24A5*, and J-L) *DHCR7*. We plotted the information for the derived allele of the lead SNP in each peak. Details of the plots are the same as in Fig. 2.

The SNP rs12913832 in *HERC2* is the major determinant of blue (versus brown) eye color through its regulatory effects on *OCA2*, and is a known target of selection un Europe [25, 1]. We detect relatively consistent selection and a persistent difference in frequency across ancestry groups (Fig. 4A-C), likely reflecting the North-South frequency gradient that has persisted from the Mesolithic to the present-day [26]. In contrast, at skin pigmentation loci *SLC45A2* and *SLC24A5*, we find consistent selection driving allele frequencies almost to fixation. Selection on skin pigmentation is thought to reflect pressure for increased vitamin D synthesis [27]. We find that selective pressure on *DHCR7* [28], which encodes a key part of the vitamin D synthesis pathway intensified in the past 100 generations, consistent with our previous findings from Britain [2] that selective pressure on vitamin D levels persisted or even intensified into the past few thousand years.

### Shared signals of selection related to immunity at the HLA and other loci

**Figure 5:**
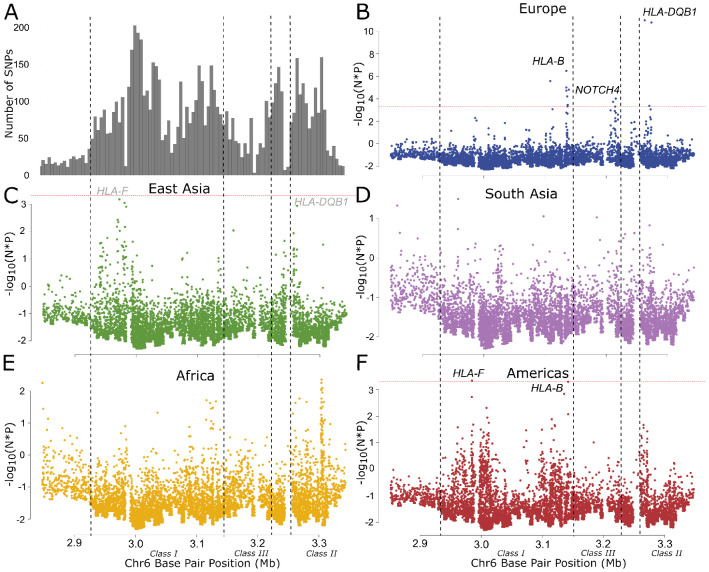
Manhattan plots corrected for SNP density focused on the MHC. We corrected for A) the distribution of SNPs across the MHC region in B) Europe, C) East Asia, D) South Asia, E) Africa, and F) the Americas. Each P-value in the manhattan plots is corrected by multiplying P by the number of SNPs in a tiled 50kb window, then the multiple testing threshold (red dashed line) corrects for the number of bins across the HLA. Dashed black boxes indicate the approximate locations of Class I, II and III HLA genes. Significant peaks are labelled in black with their nearest gene; grey labels indicate nominally-replicating sites.

The human leukocyte antigen (HLA) region on chromosome 6 is highlighted in almost every selection scan [29], due in part to the fact that it is one of the most gene-dense regions of the genome, containing hundreds of phenotypic associations including many related to immune function [30]. However, it is difficult to calibrate selection tests in the HLA region for a variety of reasons, including high levels of linkage disequilibrium; a different population history than other portions of the genome (stemming from, e.g. the effects of balancing selection), and the fact that high SNP density can lead to spurious visual peaks in genome-wide scans. Therefore, when testing for selection in the HLA, we utilized a separately calibrated empirical null distribution, and also performed a 50kb window-based Bonferroni correction to control for SNP density (Figure 5).

This approach yielded four HLA-wide significant loci: at *HLA-DQB1* in both Europe and the Americas, replicating in East Asia; *HLA-B* in Europe and the Americas; *NOTCH4* in Europe; and *HLA-F* in the Americas, replicating in East Asia. This suggests that these loci have been repeatedly targeted by selection in different populations, either because of shared selective pressures related to agriculture or other environmental transitions, or that they are common targets of selective sweeps responding to novel pathogen exposures. For example, *HLA-DQB1* has previously been identified as a target of selection in Europe [8]. Our analysis suggests that the same locus has been repeatedly targeted in different populations (Supp. Fig. S3), although we lack the resolution to tell whether it is the same haplotype. Outside the HLA, several of our other signals are also likely related to immune function including *CARD8* (Europe, replicated in East Asia and America), *MXRA7* (America) and *MIF* (America) (Supp. Fig. S1, S2; Fig. 2G-I).

### Universal stabilizing selection on female waist-hip ratio

Most human complex traits are highly polygenic. To study these traits, we extended our allele frequency-based test to detect polygenic selection. We first identify trait-associated alleles based on summary statistics from genome-wide association studies (GWAS). For each ancient and modern population, we then count the proportion of trait increasing alleles, across all variants and individuals in each population. We treat this count as though it were an allele frequency, and perform the same likelihood ratio test, conditional on admixture proportions, that we used for the per-SNP analysis.

There are several advantages to this approach. First, since it only counts observed variants, it is insensitive to genotypes missing at random. Second, we only use effect directions, not effect sizes, which can be poorly estimated in GWAS (following [7]). Finally, we can compute a well-calibrated P-value by permuting the effect directions of variants. Small P-values indicate that the distribution of trait-associated variants is very different than expected based on genome-wide admixture proportions indicating directional selection in one or more populations. Conversely, large P-values indicate that the frequencies of trait-associated variants are “too close” to the genome-wide admixture proportions, indicating that the trait is under stabilizing selection.

**Figure 6:**
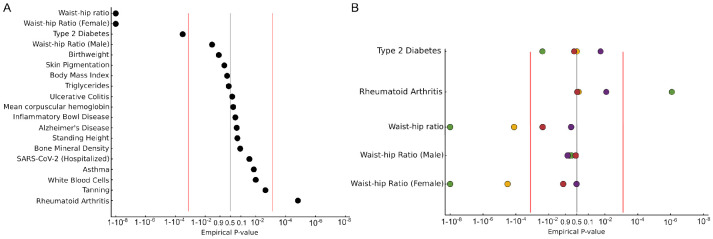
Several traits show evidence for polygenic selection. A) We tested for polygenic selection across SNPs from European-ancestry GWAS for 33 traits in jointly across 4 regions, excluding Europe to avoid the possibility of population stratification inducing false positives. B) Region-specific results for those traits with significant evidence for selection: Type 2 Diabetes, Rheumatoid Arthritis, and Waist-hip Ratio. The X-axis is plotted on a log scale, as $\text{-}log_{10}(p)+log_{10}(0.5)$ where p<0.5 and $\log_{10}(1-p)-\log_{10}(0.5)$ for p>0.5. Red lines indicate a 2-sided Bonferroni correction threshold. 19 non-redundant traits are plotted here, see Supp. Fig. S4 for the complete results.

A persistent problem with tests of polygenic selection is that they rely on variants identified in GWAS which can be confounded by population stratification, leading to false-positive signals of selection [31, 32]. One solution to this problem is to only perform selection tests on summary statistics obtained in a different population from the one on which the selection test is performed [33]. We therefore used summary statistics obtained in European populations to test for selection in all populations except Europe (Fig. 6).

Testing 33 complex traits, we find a highly significant signal of stabilizing selection on waist-hip ratio (WHR; *P* < 10^−8^), driven entirely by female waist-hip ratio (male WHR appears neutral; Fig. 6). We also find the same pattern when we test in Europe (Supp. Fig. S4; *P* < 10^−8^). WHR has been observed to be under stabilizing selection in present-day populations in the UK Biobank [34]. These consistent results suggest that strong stabilizing selection on female WHR is a fundamental aspect of human biology, presumably due to an evolutionary conflict related to reproductive biology. We also find that Type 2 Diabetes is under significant stabilizing selection, which replicates when we use East Asian T2D summary statistics in non-East Asian populations (Supp. Fig. S5, S6, Supp. Table 2). Again, the basis for this is unclear, although the genetic correlation between T2D and birth weight—a classic example of a trait under stabilizing selection due to maternal-fetal conflict—is high, which might explain the signal.

We find significant directional selection on rheumatoid arthritis (P = 1.5 × 10^−5^) and a large but not significant signal of selection on tanning response (P = 0.002), both driven by East Asia (Supp. Table 3). If we test European summary statistics in Europe (Supplementary Fig. S4) we find directional selection on skin pigmentation, bone mineral density (BMD), body mass index (BMI) and mean corpuscular hemoglobin (MCH). Skin pigmentation does seem to reflect the effect of directional selection, as shown by other lines of evidence. The fact that we do not find it when testing non-European populations likely reflects the population-specificity of the loci driving adaptation in Europe. While BMD, BMI and MCH are all plausible targets of directional selection, we cannot rule out that these signals result from population stratification.

## Discussion

Our results highlight several key aspects of recent natural selection in humans. First, we confirm that classic (i.e. strong, complete) selective sweeps are rare in recent human evolution [35]. Second, for many of the partial selective sweeps that do exist, the strength of selection varies with time, space and ancestry. Third, many signals of selection are shared between populations. In some cases (e.g. *FADS1*) the same haplotype was selected in different populations, while in others (e.g. *ADH1B*, perhaps *HLA-DQB1*), different haplotype at the same locus were selected. Similarly, the strongest signals of polygenic selection we detected—of stabilizing selection on WHR and T2D—are largely shared across populations.

These shared signals of selection may reflect adaptation to shared selective pressures, for example the adoption and development of agriculture independently in many parts of the world. For example, the strongest signals of selection at individual loci (*LCT, ADH1B, FADS1*) are all plausibly related to the consumption of agricultural products. Denser time series will allow greater temporal resolution on the timing of selection and allow us to address the question of how tightly selection at different loci was related to agriculture and other environmental changes. Shared signals may also reflect the fact that some loci might be repeatedly targeted by sweeps. For example, genes involved in immunity might experience repeated sweeps, or parallel sweeps in different populations, even if different pathogens are involved. Finally, some shared signals might reflect imperfect modeling. For example, while the European pigmentation locus *SLC24A5* nominally replicates in South Asia, that is likely due to recent shared ancestry between Europe and South Asia which our approach does not model correctly due to the limited South Asian ancient DNA sample size, rather than a real signal of independent selection in South Asia. Indeed, time series analysis shows the selection coefficient s is not significantly different from zero at any point in time after controlling for ancestry (Supp. Fig. S2).

Skin pigmentation in Europe is well-known to have been under selection, probably in response to differing UV exposure at higher latitudes and the downstream effects on Vitamin D biosynthesis [27, 36]. We confirm this result, but we do not see such a clear signal in other populations. For example, in East Asia, none of the genome-wide significant loci are clearly related to pigmentation and we see no polygenic signal of selection on skin pigmentation using either European or East Asian summary statistics. We do see a suggestive signal of polygenic selection on tanning response, raising the hypothesis that adaptation to latitude in East Asia focused on plastic response rather than constitutive skin pigmentation as in Europe.

The immune system is another key target of selection. While some HLA loci seem to be adaptive in multiple regions, others appear to be population-specific, as are several of the non-HLA immune loci we found. This is also reflected in the significant directional selection on loci influencing Rheumatoid Arthritis in East Asia. RA is an autoimmune disease with a large role played by inflammatory pathway including HLA genes [37]. While RA itself is likely not the source of selective pressure, it is an example of a clinical trait being influenced by evolution; pro-inflammatory signals may be beneficial in the case of an infection, but cause problems in other contexts.

We find that female waist-hip ratio (WHR) is consistently under stabilizing selection. However, empirically many populations have higher female WHR than might be expected given the fact that lower female WHR is generally associated with increased fertility. The advantage of a higher WHR is unclear although it may lead to better health in some contexts (e.g. in times of resource scarcity) [38]. The sex-specific selective pressure probably explains why the genetic correlation between sexes is lower for WHR than for almost any other complex trait [39, 40].

The main limitation of our study is that we are still relatively underpowered, particularly outside Europe. Even in Europe, power drops off rapidly for selection coefficients between 1-2%. While the number of loci we detect is broadly consistent with other approaches using comparable significance thresholds [1, 3, 5-7], it seems likely that there were many more variants under selection, with selection coefficients less than 1%. Indeed, some other scans (e.g. [8]) find exactly that (Supplementary Note C). However, the questions of how accurately those sweeps can be identified, how much they are shared across populations, and how much they target *de novo* or standing variation remain unresolved.

## Materials and Methods

### A variant-level test for selection

To test for selection while taking into account admixture history, we explicitly model relationships between ancient and modern populations (Supp. Fig. S7). Specifically, we use admixture proportions to calculate the expected allele frequencies for a variant, and test whether observed frequencies match that expectation [1]. Unlike the previous iteration of this method, we infer admixture proportions in both ancient and modern populations relaxing the assumption that we have samples from unadmixed source populations and more accurately reflecting the fact that ancient populations were themselves products of admixture of previous peoples.

We quantify these relationships in a likelihood framework. The log-likelihood for a given variant under the alternative hypothesis that allele frequencies across populations are unconstrained is

$$\ell_{1}=\sum_{i=1}^{A+B}x_{i}\;\log(f_{i})+(N_{i}-x_{i})\;\log(1-f_{i})$$

where A and B are the number of ancient and present-day populations respectively, $x_{i}$, $N_{i}$, and $f_{i}=x_{i}∕N_{i}$ are the observed alternate allele count, total allele counts and allele frequency for the variant in population i. To obtain the likelihood under the null hypothesis that allele frequencies are constrained, $\ell_{0}$, we calculate the expected frequencies in our set of populations as:

$$(f_{1},\cdots ,f_{A+B})=M\cdot Q$$

where

$$M=\left[\begin{array}{ccc} P_{1}^{1} & \cdots & P_{1}^{k}\\ \vdots & \; & \vdots \\ P_{A}^{1} & \cdots & P_{A}^{k}\\ P_{A+1}^{1} & \cdots & P_{A+1}^{k}\\ \vdots & \; & \vdots \\ P_{A+B}^{1} & \cdots & P_{A+B}^{k} \end{array}\right]$$

and

$$Q=(q_{1},\cdots ,q_{k}),$$

where M is a (A+B)×k matrix containing the admixture proportions for the A+B ancient and present-day populations for each of the k source populations, and Q is a vector of length k containing the allele frequencies of the variant in each of the source populations. We estimate M using ADMIXTURE v1.3.0 [12], after conducting LD pruning (200kb windows, 25-variant step, $r^{2}>0.4$). For the non-European samples, we ran ADMIXTURE in unconstrained mode, across a range of source population numbers from K=2 to K=6 and used the K with the minimum cross-validation error. (Within Europe we fixed K=3 because of well-established evidence for three source populations.) Conditional on M, we fit Q to maximize

$$\ell_{0}=\sum_{i=1}^{A+B}x_{i}\;\log(f_{i})+(N_{i}-x_{i})\;\log(1-f_{i}),$$

thereby obtaining both the null likelihood and the expected frequencies in our source populations. Q is equivalent to the Q-matrix generated by ADMIXTURE but we fit it numerically for consistency with the calculation of the alternative likelihood.


$$\ell_{0}=\sum_{i=1}^{A+B}x_{i}\;\log({\hat{f}}_{i})+(N_{i}-x_{i})\;\log(1-{\hat{f}}_{i}).$$

The value of $2|\ell_{1}-\ell_{0}|$ follows a $\chi^{2}$ distribution with A+B−k degrees of freedom. We additionally controlled for inflation due to genetic drift or unmodeled ancestry variation using genomic control [41], dividing the $\chi^{2}$ statistics by a constant so that the median statistic matches the theoretical value, and calculate a P-value based on the $\chi_{A+B-k}^{2}$ distribution.

We ran this scan for 1,150,640 variants captured by the 1240k reagent commonly used for ancient DNA data [10, 11]. Because we were combining genetic data across data sets, technical artifacts from one dataset could drive false positives in this scan. We therefore filtered all variants with FDR > 0.1 by requiring that at least one additional variant within 200kb and high LD ($r^{2}>0.5$) also pass that FDR threshold. We calculated LD for this purpose using the 1kG populations corresponding to each region. We then focused on signals passing a genome-wide Bonferroni multiple testing correction with *p* < 5 × 10^−8^. We plotted individual significant signals using LocusZoom [42].

For each significant signal, we tested for replication in the other regions both of the lead variant and of the locus more generally. To test if the lead variant replicated we tested for all lead variants in a specific region by examining their P-values in each other region, using a Bonferroni correction for the number of lead variants in that region. To test for the general locus, we identified the minimum p-value within a megabase of the lead SNP, then used a Bonferroni correction for the number of SNPs tested in the window to determine replication.

### Population samples and admixture models

We tested for selection in 5 regions that had genetic representation of both ancient and present-day populations: Africa, East Asia, South Asia, Europe and Central/South America (Fig. 1A). Most data were obtained from the Allen Ancient DNA Resource (AADR) [43], with additional samples from the HGDP [44] where necessary. For each region, we projected samples into PCA space constructed using samples genotyped on the Human Origins array from the same regions, then identified groups of genetically-similar ancient samples that encompassed the present-day populations. We defined those groups by drawing polygons around them in PCA space. As most ancient samples were pseudohaploid, we used pseudohaploid versions of samples from the 1000 Genomes project, reported in the AADR.

In Africa, we included the Yoruba (YRI; N=101) and Luhya (LWK; N=101) populations from the Thousand Genomes Project (1kG) [45], as well as Biaka from the Human Genome Diversity Project (HGDP;BIAK; N=21) [44]. We identified 3 distinct ancient groups corresponding to Central Africa (CENT; N=33), East Africa (EAST; N=149), and those genetically similar to present-day Bantu speakers (BANT; N=15) (Supp. Fig. S8). We used K=3 for ADMIXTURE modeling. The ancient samples used here were originally published in [46-53]. To construct the PCA we used Human Origins samples published in [52, 54-58].

For East Asia, we represented present-day populations with five from 1kG: Han Chinese from Beijing (CHB; N=103) and Southern China (CHS; N=106), Chinese Dai (CDX; N=99), Japanese (JPT; N=104), and Vietnamese (KHV; N=97). We had 3 ancient groups corresponding to Northern China (NOR; N=103), Southern China (SOU; N=107), and the Jomon in Japan (JOM; N=24), and used K=3 for ADMIXTURE (Supp. Fig. S9). The ancient samples used here were originally published in [59-72]. To construct the PCA we used Human Origins samples published in [54, 56, 73, 71].

For South Asia we used Gujarati (GIH; N=105), Punjabi (PJL; N=96), Telugu (ITU; N=103), Tamil (STU; N=99), and Bengali (BEB; N=85) populations from 1kG, and kept the ancient samples in 1 group (ANCS; N =121). We used k=2 for ADMIXTURE (Supp. Fig. S10). The ancient samples used here were originally published in [74, 75]. To construct the PCA we used Human Origins samples published in [54, 56, 71, 76].

For present-day European populations we used Italian Toscani (TSI; N=108), Iberian (IBS; N=103), British (GBR; N=92) and Northwestern Europeans (CEU; N=99) from 1kG, and split the ancient samples into 5 groups. These were hunter-gatherers (HG; N=157), early farmers (EF; N=605), admixed early farmers (EFAD; N=892), northern Bronze Age (BAN; N=2113), and southern Bronze Age (BAS; N=1000). We used k=3 for ADMIXTURE (Supp. Fig. S11). The ancient samples used here were originally published in [1, 11, 26, 60, 75, 77-156]. To construct the PCA we used Human Origins samples published in [54, 56, 57, 73, 157].

In Central/South America (shortened to ‘Americas’ in the manuscript), we used 1kG Peruvians (PEL; N=69) and HGDP Mayans (MAYA; N=18) for our present-day populations, and included IBS to represent the European ancestry present in many present-day Americans. We left the ancient samples in one group (ANCA; N=216), and used k=2 for ADMIXTURE (Supp. Fig. S12). The ancient samples used here were originally published in [158-170]. To construct the PCA we used Human Origins samples published in [54, 56].

### Power Simulations

To estimate the power of our analysis, we conducted Wright-Fisher simulations of selection for each of the five regions (Supp. Note A). For one variant in one population, we start the simulation at the observed frequency, then draw the alleles in the next generation from a binomial distribution based on a population of size $N_{e}$ with a selection probability ($p_{sel}$) of

$$p_{sel}=\frac{f(1+s)}{1+fs}$$

where f is the frequency in the previous generation, and s is the strength of selection. For each population, we randomly selected 1000 variants (constrained to MAF > 0.05 in that population), then simulated their allele frequency trajectories for 0, 10, 20, 50, and 100 generations, under selection strengths varying from s=0 to s=0.1. We then conducted the selection scan as for the real data, swapping in the simulated frequency for that population for each SNP, and using the same genomic control and multiple testing corrections as for the real scan.

### Time series analysis

To analyze time series of allele frequencies while accounting for population continuity and admixture, we extended our previous approach [2] to incorporate individual-specific admixture loadings. Following the notation in that paper, let $f_{tk}$ be the frequency of the derived allele in population k at time t generations into the past, and similarly let $s_{tk}$ be the strength of selection and $N_{tk}$ the effective population size. Given these, we assume as in the original paper that $f_{t-1,k}=F_{t-1,k}∕[2N_{t-1,k}]$, where

(1) $$F_{t-1,k}\;|\;f_{t},s_{t}∼\text{Binomial}(2N_{t-1,k},f_{tk}^{\prime }(s_{tk}))$$

and $f_{tk}^{\prime }(s_{tk})$ is the allele frequency in population k immediately after mating (see equation (2) in [2].)

Suppose that we sample $n_{t}$ individuals at time t. The genotype of sample $i\leq n_{t}$ at time t is denoted $a_{ti}\in \{0,1\}$, where without loss of generality we assume that each individual is haploid. (Diploid individuals can be treated as two haploid individuals.) The population that this allele was inherited from is denoted $z_{ti}\in \{1,\ldots ,K\}$. Hence,

(2) $$a_{ti}\;|\;z_{ti}=k∼\text{Bernoulli}(f_{tk}).$$

While we do not observe $z_{ti}$ directly, we do assume knowledge of the overall proportion of alleles that were inherited from population k, denoted $q_{tik}$. Hence, $Q_{t}=(q_{tik})\in {\left[0,1\right]}^{n_{t}\times K}$ is exactly the loading matrix obtained by running ADMIXTURE [12] on the samples.

In the single population case, our original bmws model [2] worked by modeling the single-population allele frequency $f_{t}$ as a mixture of beta distributions, with atoms at $f_{t}=0$ and $f_{t}=1$. However, it is challenging to extend this approach to the multi-population setting, because the joint distribution of $f_{t}$ is a mixture of ${\left(M+2\right)}^{K}$ components, representing all possible combinations of the M beta components and the two atoms from each population.

To avoid this combinatorial explosion, we instead used particle filtering [171], whereby a discrete set of atoms ${𝒫}_{t}\subset {\left\{0,\ldots ,2N\right\}}^{K}$, $|{𝒫}_{t}|=P$ are used to approximate the distribution of $f_{t}$ at each time point. (For simplicity we assumed that the effective population size N is known and constant over time and populations, but this is not essential.) Each particle $p_{tj}\in {𝒫}_{t}$, j=1,…,P represents the number of derived alleles in each of the K populations, and the particles are updated over time by incorporating the information from $n_{t}$ and $Q_{t}$ at each time point. Each particle has an associated weight $\omega_{p_{t}}$, and the distribution of $f_{t}$ is approximated by the weighted empirical distribution of the particles:

$$f_{t}∼\sum_{p_{t}\in {𝒫}_{t}}\omega_{p_{t}}\delta_{p_{t}∕(2N)}.$$

The particle filter works by iterating over time points t=T, T−1,…,1, transitioning the particles from time t to time t−1 by incorporating the effects of genetic drift and selection, and then updating the weights $\omega_{p_{t+1}}$ to $\omega_{p_{t}}$ by incorporating the information from the observed data. Concretely, given ${𝒫}_{t+1}$, we form ${𝒫}_{t}$ by transitioning each particle according to the model described above:

$$p_{t}\;|\;p_{t+1}∼\text{Binomial}(2N,p_{t+1}^{\prime }),$$

where $p_{t+1}^{\prime }$ is the allele frequency in each population immediately after mating and selection when the allele counts are given by $p_{t+1}$.

Next, we incorporate the information from the data to update the weights. The likelihood of the data conditional on population membership $z_{ti}$ is given by (2) above. Therefore,

$$\omega_{p_{t+1}}\propto \omega_{p_{t}}\prod_{i=1}^{n_{t}}f_{t,z_{t,i}}.$$

Repeating in this way for t=T, T−1,…,1, we obtain a set of particles ${𝒫}_{0}$ and weights $\omega_{p_{0}}$ that approximate the distribution of $f_{0}$. Samples from the posterior distribution $f_{0:T}\;|\;a_{1:T}$, $Z_{1:T}$ can be obtained by sampling a particle $p_{0}$ from ${𝒫}_{0}$ according to the weights $\omega_{p_{0}}$, and then tracing back the ancestry of that particle through time.

### Gibbs sampler for selection and allele frequency trajectories

To sample from the posterior distribution of selection coefficients $s_{1:T}$ we use Gibbs sampling. We iteratively sample from the following conditional distributions:

(3) $$f_{0:T}\;|\;a_{1:T},Z_{1:T},s_{1:T}$$

(4) $$Z_{1:T}\;|\;a_{1:T},f_{0:T},Q_{1:T}$$

(5) $$s_{1:T}\;|\;f_{0:T}$$

Sampling from the distribution (3) is done using the particle filter described above. Sampling from the distribution (4) is straightforward, as the $z_{ti}$ are independent given $f_{0:T}$ and $Q_{1:T}$. Specifically, we have

$$P(z_{ti}=k\;|\;a_{ti},f_{0:T},Q_{1:T})\propto q_{tik}f_{tk}^{a_{ti}}{\left(1-f_{tk}\right)}^{1-a_{ti}}.$$

Finally, to sample $s_{1:T}$ (5), we leverage that $s_{t}$ is independent of all other quantities conditional on $\left(f_{t},f_{t-1}\right)$. The conditional likelihood of $s_{t}$ is given by (1) above, multiplied by the prior on $s_{t}$ (see next section). Viewed as a function of $s_{t}$, the density (1) does not correspond to any known distribution, so we approximately sample from it using the Metropolis-adjusted Langevin algorithm [172].

### Regularization

In the original bmws method, allele frequency trajectories were regularized via the smoothing penalty $\alpha \sum_{t=2}^{T}{\left(s_{t}-s_{t-1}\right)}^{2}$ to prevent overfitting, where α is a tuning parameter controlling the amount of regularity. As α→∞, the estimate s shrinks to a constant value, implying no time-varying selection.

Here we incorporate an additional term that encourages between-population similarity in these trajectories, in recognition of the fact selective pressures within and among the various ancestral populations likely covaried, at least partially. To model this, we add an additional penalty term of the form

$$\beta \sum_{t=1}^{T}\sum_{i\neq j}{\left(s_{ti}-s_{tj}\right)}^{2}$$

which penalizes the pairwise differences between the selection strength at each time point. As β→∞ the model estimates shrink to a single common trajectory (note that this may still be time-varying, depending on α), implying that selection does not vary based on ancestry background.

To enable the method to adapt to the level of signal in the data, we adopted a fully Bayesian approach, placing hyperpriors on α and β. Specifically, we used independent Gamma priors $\alpha ∼\text{Gamma}(a_{\alpha },b_{\alpha })$ and $\beta ∼\text{Gamma}(a_{\beta },b_{\beta })$, where $a_{\alpha ,\beta }$ and $b_{\alpha ,\beta }$ are hyperparameters. Then, α and β are sampled as part of the Gibbs sampler described above according to standard updates:

$$\alpha \;|\;s∼\text{Gamma}\left(a_{\alpha }+\frac{TK}{2},b_{\alpha }+\frac{1}{2}\sum_{t=1}^{T}\sum_{k=1}^{K}{\left(s_{tk}-s_{t-1,k}\right)}^{2}\right)\;\beta \;|\;s∼\text{Gamma}\left(a_{\beta }+\frac{TK(K-1)}{2},b_{\beta }+\frac{1}{2}\sum_{t=1}^{T}\sum_{i\neq j}{\left(s_{ti}-s_{tj}\right)}^{2}\right).$$

We chose these hyperparameters by comparing the log likelihoods across multiple different alphas for each variant in each population, and chose one alpha that maximized the average log likelihood (or was very close to the maximum) for all regions (Supp. Fig. S13). For the results presented here, we used α=2.0 and β=α∕100.

### Time Series Simulations

To characterise the performance of bmws-admix we developed a simulation framework allowing us to mimic observed data to varying degrees (Supplementary Note D). We simulated our populations with k=3 ancestry components, since that was the number we used for our best-powered selection scans. We then used a Wright-Fisher framework for each ancestry component independently, allowing for variable s, then sampled admixed individuals at each time point to get the overall allele frequencies.

### Test for polygenic selection

To test for selection across multiple variants contributing to a polygenic trait, we adapted the same pipeline used for the locus-based test. Instead of counting the number of alternate alleles for a single variant across populations, we counted the proportion of trait-increasing alleles across all significant variants identified in a genome-wide association study (GWAS). For population i, $N_{i}$ is the total number of non-missing (pseudohaploid) genotypes across all individuals and significant variants, and $x_{i}$ is the total number of genotypes with the trait-increasing allele. The likelihood calculation is then exactly the same as for the single variant test, but we calculate P-values based on a null distribution obtained by permuting the direction of effects and recalculating the likelihood, rather than based on the $\chi^{2}$ distribution.

We did this for 38 biometric and disease traits analysed in European ancestry individuals: Alzheimer’s Disease [173], Asthma [174], Covid-19 (3 degrees of severity; Freeze 7 of the HGI) [175], Coronary Artery Disease (CAD) [176], Irritable Bowel Disease, ulcerative colitis (UC), Crohn’s Disease [177], HDL levels, LDL levels, triglycerides [178], Rheumatoid Arthritis (RA) [179], Type II Diabetes [180], and Waist-hip ratio (all, male, and female) [181], estimated Glomerular Filtration Rate [182]. For the remaining traits we used summary statistics calculated in European-ancestry individuals from the UKBB by the Neale Lab (http://www.nealelab.is/uk-biobank/): Mean corpuscular hemoglobin (MCH), Mean corpuscular volume (MCV), counts of blood cells (platelets, red and white blood cells), Bone Mineral Density, Sitting and Standing Height, Waist Circumference, Hip Circumference, Skin Pigmentation, Tanning, Body Mass Index (BMI), Birth weight and Age at Menopause.

For each trait, we filtered for variants present on the 1240k array, and clumped the association signals using Plink [183]. Clumped SNPs were required to be less than 200 kb apart and have $r^{2}>0.4$. We then counted trait-increasing alleles across all lead 1240k variants with $p<1\times 1^{-8}$ for each trait. We calculated P-values by permuting the effect direction of each variant included 1,000,000 times, thereby changing which counts were contributing to the trait-increasing alleles. The underlying allele frequencies remained unchanged. We identified traits there showed significant directional and stabilizing selection by calculating 2-sided Bonferroni multiple testing thresholds for N traits as 0.025∕N for directional, and (1−0.025)∕N for stabilizing selection, where N is the number of traits. This choice tends towards being conservative, as we corrected for all traits tested, regardless of their redundancy of others (e.g. we included 3 different metrics of SARS-CoV-2 occurrence).

We also repeated this analysis using 19 traits from GWAS in East Asian-ancestry cohorts: Bone Mineral Density, Body Mass Index, HDL, LDL, Triglyceride levels, Standing Height, Platelet, Red Blood Cell, White Blood Cell counts, and Waist-hip ratio [184]; Asthma, Mean Corpuscular Hemoglobin, Mean corpuscular Volume, Rheumatoid Arthritis, Ulcerative Colitis [185]; Coronary Artery Disease, estimated Glomerular Filtration Rate, Age at Menopause [186]; Type II Diabetes [187]; skin pigmentation [188]. In many cases these GWAS were less powered than the European versions, so for 4 traits with fewer than 40 hits at the stricter threshold (Menopause, UC, Skin Pigmentation, WHR) we lowered the genome-wide significance threshold to 1 × 1^−6^ to include enough variants for the selection scan.

### Comparison with other selection scans

To determine how our results compare to those of other selection scans, we compared our European scan (with the highest power and most other scans to compare) to 4 other scans: [1, 3, 6, 8]. For each set of published scores we intersected the variants tested with those in our scan. Because Kerner et al. only published a list of significant tag SNPs, we also pruned the significant results of the other three, as well as ours, then calculated the enrichment of the hits in each scan in our top N% of peaks. We pruned using Plink’s clumping function with a window of 1Mb and $r^{2}>0.4$ [183]. See Supplementary Text for further details and discussion.

## Supplementary Material

Supplement 1
