# Supplementary material for: Global patterns of natural selection inferred using ancient DNA
Source: bioRxiv. 2026 Jan 8:2026.01.07.697984. Preprint. [Version 1] doi: 10.64898/2026.01.07.697984 (PMC12803250; doi:10.64898/2026.01.07.697984)
Supplement: Supplement 1 [file media-1.pdf]

Supplementary Text

A Power analyses

We conducted power simulations in order to better understand what could be driving the differences between the number of significant hits in different regions for the AF-based test. While variable sample sizes would likely influence power, the varying demographic histories and our ability to model them using ADMIXTURE would also be expected to have an influence. We therefore simulated selection using the Wright-Fisher model for random variants in each population in all five regions. We required the MAF of each variant is greater than 0.05, and for each variant set the initial frequency at observed frequency. We did this across a range of selection strengths (0-0.1), and for 1000 random variants in each population, then combined for each region to see how many came up as significant.

As expected, power to detect selection increased with the strength of selection, and was higher in regions with higher sample size (Supp. Fig. A1). Interestingly, power does indeed seem to decrease with more complicated demography, with the maximum in Africa being about half that in the Americas, despite similar sample sizes. It is also notable that, despite the much larger sample size, even Europe asymptotes at about 80% power. This seems to be primarily driven by how much of the allele frequency trajectory we observe; power is lower for variants that are already at high frequencies at the start of the time series.

Together, this suggests that the differences in apparent selection between regions is likely explained entirely by power differences, influenced both by overall sample size and by the extent to which demographic history captured by the ADMIXTURE analysis of the samples. To illustrate this, we ran two analyses downsampling the ancient European samples– A) randomly downsampling the whole region to match the overall sample size we had in East Asia, and B) downsampling the individual populations so that they matched the population sizes in East Asia. For A) this resulted in 236 individuals, with 6 HG, 41 EF, 45 EF-ADMIX, 98 BA-N, and 46 BA-S samples, while B had 425 individuals with 23 HG, 94 EF, 103 EF-ADMIX, 102 BA-N and 103 BA-S. In both cases we retained the 4 present-day populations, each with approximately 100 samples.

In Analysis A, no loci passed an FDR multiple testing correction, despite the overall sample size being identical to that of East Asia where we found multiple significant loci (Supp. Fig. A2A). However, when we increased it to similarly represent each relevant population (the smallest having 23 samples, the rest around 100 each), we acquire 2 Bonferroni-significant peaks at *LCT* and *TBC1D13*, with *HERC2* also passing FDR (Supp. Fig. A2B). Together with the variation in number of hits across regions, despite the similar ancient sample sizes in the non-European regions (100-200 each), this suggests that raw sample size is only useful if the additional samples correctly represent allele frequencies in the ancient groups, and if they allow ADMIXTURE to infer ancestry clusters that somewhat correspond to actual source ancestry.

We believe this is why the scans in East Asia and the Americas were more successful than in South Asia and Africa, despite similar sample sizes. In both former cases the ADMIXTURE and samples were able to capture known demographic history, reflecting specific known admixture dynamics (northern and southern mainland, and Japan in East Asia, more recent European admixture in the Americas). On the other hand South Asia only had ancient samples from the northern part of the region, and Africa has a deep and complex genomic history genetic history that is not well represented with a limited sample size compared to populations that were subject to the out-of-Africa bottleneck. As we increase the sample sizes and our knowledge of demographic history in each region, we will be better able to represent ancient groups (e.g. separating out Central and South American populations).

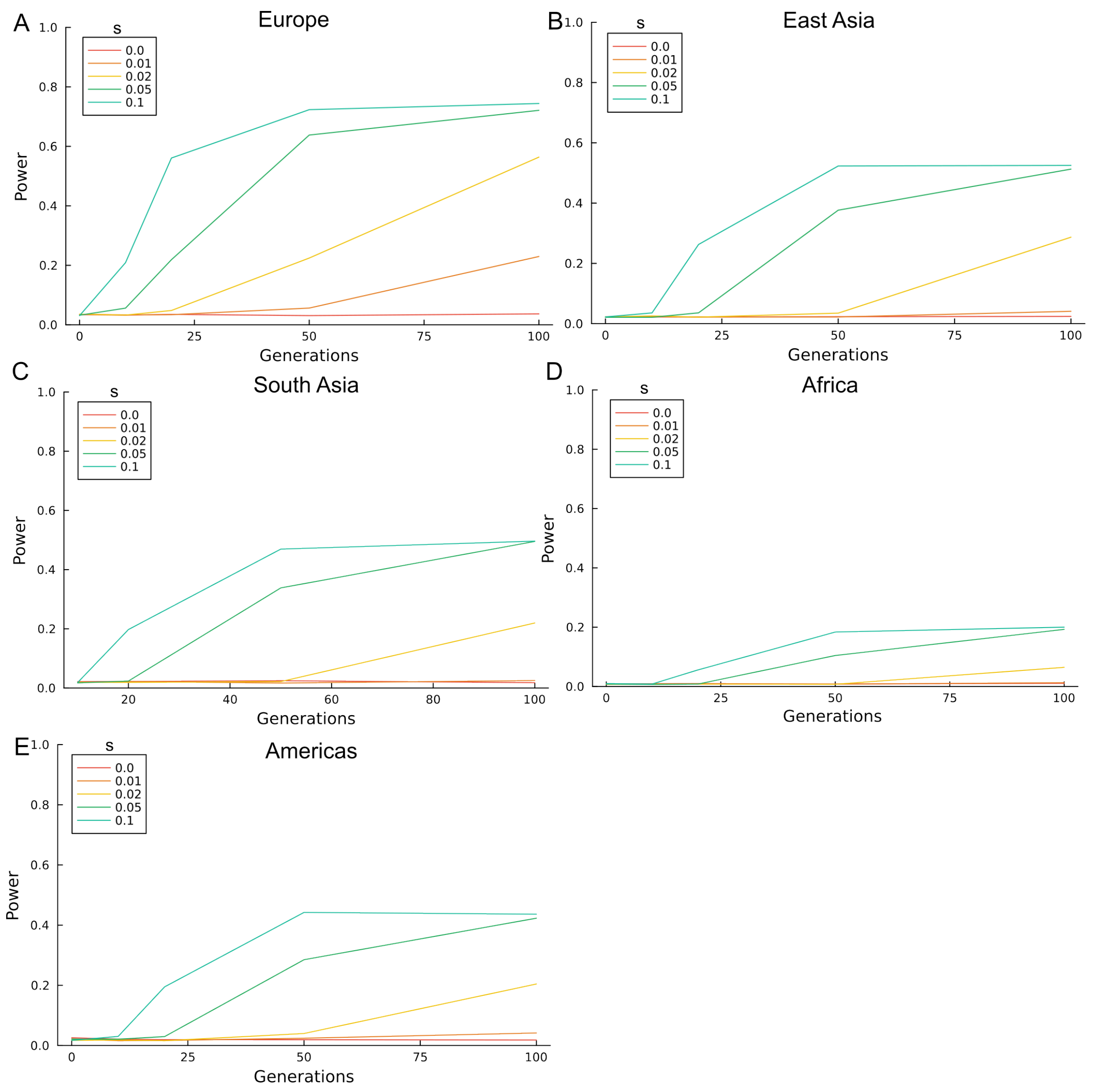

Supplementary Figure A1: Power curves based on Wright-Fisher simulations.
We simulated selection for 1000 random variants in each population in A) Europe, B) East Asia, C) South Asia, D) Africa, E) the Americas. We did this for s = 0, 0.01, 0.02, 0.05, 0.1, and from 0 to 100 generations.

B Comparison to other European selection scans

While this is the first aDNA-based scan in most of the regions we study here, Europe has much larger availability of aDNA and correspondingly has been studied for selection in similar ways before. We therefore compared our results to both the scan run with the original method modified here [1] and with a recent alternative allele frequency approach [2]. We also compared our results with a study based on local ancestry [3], and with an ABC simulation-based method [4].

In intersecting our results with these other scans, we found broad agreement, with the top hits of each other scan being enriched in our top hits (Supp. Fig. B1A). Furthermore, this enrichment increased as we were stricter in which hits were considered the ”top” ones. It was also notable that the consistently most-enriched results were those from Mathieson 2015, the direct predecessor to this study, while the Kerner et al. with the most distinct methodology were the least enriched (although we lacked access to their full summary statistics, so had to limit ourselves to just the variants they identified as significant).

Despite the broad agreement in patterns of selection across loci, we found the drastic difference in

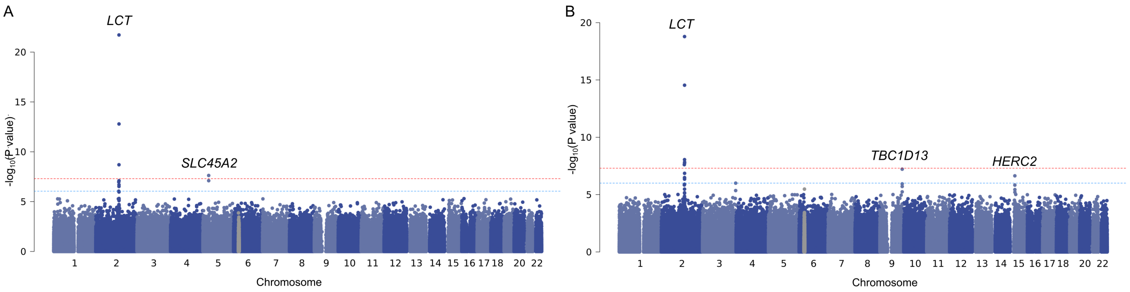

Supplementary Figure A2: European locus-based scans with downsampled ancient groups. A) Downsampling randomly to 236 ancient individuals overall. B) Downsampling such that the smallest ancient group had 23 samples, while the other four each had approximately 100.

number of significant peaks identified in these studies (ranging from a dozen to hundreds) striking. We particularly focused on Akbari et al., which reported 347 independent significant loci. While some of that increase is due to sample size (about 3000 additional ancient samples), the main factor is their less-stringent genome-wide significance threshold, determined by identifying enrichment of selection signals in GWAS hits, which effectively does half the amount of correction in genomic-control than we implement here. Indeed, if we amend our lambda correction to a similar level we identify 1050 independent loci (Supp. Fig. B1B).

C Discussion of other significant loci

ABI3BP; Europe. Lead SNP is in an intron of *ABI3BP*, and is primarily an eQTL for *PDCL3P4*, a pseudogene. *ABI3BP* itself is not well characterized.

CABP7; Europe. The lead SNP is in an intron of *ZMAT5*, and is a convincing eQTL for *NIPSNAP1*, a mitochondrial protein with possible involvement in inflammation [5, 6]. It is also an eQTL for *MTMR3*, which is near a GWAS hit for IBS [7].

CARD8; Europe, replicated in the Americas and East Asia. This haplotype is an eQTL for many genes in the locus, in many different tissues, particularly *ODAD1*, and an sQTL for *CARD8* [8]. *ODAD1* is tied to primary ciliary dyskinesia (recurrent lung infections), and is primarily expressed in the lung [9, 10]. *CARD8* is a inflammasome-forming gene with human-specific HIV-sensing functionality [11], as well as wider involvement in inflammatory pathways via different isoforms [12].

CFAP58; the Americas. The lead SNP is an eQTL for *GSTO1*. *CFAP58* is involved in sperm morphology and development [13], while *GSTO1* is involved in inflammatory pathways and has been associated with severity of symptoms in response to high arsenic exposure [14–17].

EGFLAM; Europe, replicated in the Americas. The lead SNP is in an intron of *EGFLAM*, but isn't an eQTL. There are some nearby immune GWAS hits [7], but no obvious functional connection.

HEATR6; East Asia. While the lead variant at *HEATR6*, rs2333656, is an eQTL for *HEATR6* and several nearby RNA genes, we do not believe *HEATR6* is a likely target of selection, given its broad expression and lack of known function. However, another variant on the same haplotype (rs345166, $p = 4.1 \times 10^{-}7$), is an eQTL for *CA4*, a zinc-metalloenzyme highly expressed in the lung, colon, adipocytes, and whole blood [8, 18]. Along with other members of the CA gene family, *CA4* catalyzes the hydration of carbon dioxide and may play a role enhancing wider metabolism via interactions with *MCT* transporters [19, 20].

HS3ST1; Europe. This peak is 650kb from the gene, not near anything else, and isn't an eQTL. *HS3ST1* is important for heparan sulfate biosynthesis [21]. There are nearby GWAS associations with Alzheimer's Disease, ALS, HDL cholesterol, and BMI [7].

IRX2; Europe. This SNP is a long way from both *IRX1* and *IRX2*, with nothing particular around it. There are some GWAS associations with Waist and hip circumference and height [7], but no obvious functional explanation.

KCNIP1; Europe. The lead SNP is in an intron of *KCNIP1*, but is not an eQTL for anything. There is a nearby GWAS hit for HIV viral load and some ALL associations, but no obvious link to this variant or gene [7]. *KCNIP1* may be involved in Type II Diabetes [22, 23].

KHDRBS3; Europe, replicated in the Americas. The lead SNP is not an eQTL, and is nearly a megabase from this gene, which doesn't have a big depth of literature about it. There is a Bone Mineral Density GWAS haplotype in the general vicinity, but not much else [7].

MIF; Americas. Macrophage migration inhibitory factor [24]. The lead SNP is an eQTL for the antisense transcript of *MIF* [8], and we hypothesize that it may regulate *MIF* itself, at least in some conditions (GTEx does not test expression under immune challenge, for example.).

MXRA7; The derived allele of the lead SNP at *MXRA7* was less frequent than expected, primarily in the British population. This haplotype is an eQTL for several genes in the region, though *MXRA7* is significant in the most tissues. The derived allele actually decreases expression of the gene in most tissues, though not all. The gene is involved in macrophage differentiation [25], so potentially associated with host defense.

NAT2; Europe. Previously identified locus [26–28], although a different haplotype than previously identified. although the mechanism of selection is unknown. NAT2 interacts with many compounds found in both diet and the environment [29].

NUP85; Europe. The peak at *NUP85*, which is involved in viral response and immune cell migration [30–32] is actually the same signal we previously identified in a time-series-based scan of selection on gene expression in ancient Britons [33], though our results here suggest this is a more general European signal.

ROR2; Europe, replicated in Africa. The lead SNP is an eQTL for *ROR2* and *SPTLC1*. *ROR2* is involved in cartilage and growth plate development, as well as Brachydachtyly type B [34]. There is a nearby GWAS association for height, and BMI [7]. *SPTLC1* is a key enzyme in sphingolipid biosynthesis [35]. This locus has been previously seen in a PBS-based selection scan in Khoe-San [36].

SCN2A; East Asia, replicated in South Asia. This haplotype is an eQTL for *SCN2A* in cerebellum tissues, and *COBLL1* in testis, as well as *CSRNP1* in skin [8]. *SCN2A* is associated with GWAS traits related to autism and seizures, as well as alcohol use disorder, while *COBLL1* has GWAS hits related to WHR, BMI and lipoproteins, and has variants that modulate obesity [7, 37, 38], suggesting it is more likely to affect fitness in a population-specific way.

SDC4; South America. The lead SNP is in an intron of *SDC4*, and is an eQTL for *SDC4*, *SYS1* and *WFDC5* [8]. This locus also contains the WFDC gene cluster, which has been previously identified in targeted positive and balancing selection scans in African and European populations and in Chimpanzees and contains genes involved in both innate immunity and fertility [39–41].

SLC22A4; Europe. Previously identified signal [42, 1, 3] near the ergothioneine transporter gene, but also associated with IBD and asthma so potentially related to either diet or immunity.

UFM1; Europe. The only eQTL on this haplotype is for *TRPC4*, a cation channel with roles in many processes and is particularly highly expressed in the uterus [8]. *UFM1* is a modifier similar to ubiquitin, and is tied to cancer progression [43].

ZSCAN31; Europe. The lead SNP is in the intron of this gene, and is an eQTL and sQTL for several nearby genes in many tissues. The nearest GWAS hits are for MDD, Covid-19 and staph aureus infections, pork/oily fish diet, and gas reflux [7]. Most of the genes in the region are understudied zinc fingers, so a functional tie to any of these is not possible. This peak is just outside the MHC region.

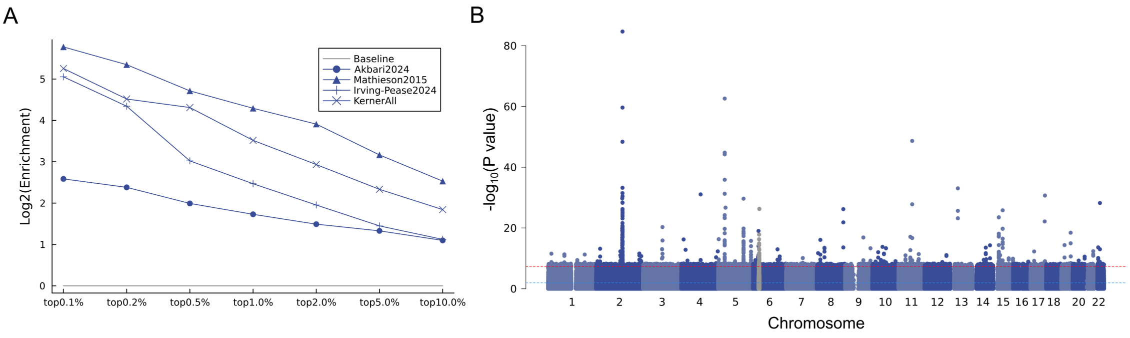

Supplementary Figure B1: Comparison with other European aDNA selection scans.
A) Enrichment of top results from other scans in our top N% hits in Europe. B) Results of our European scan using a GC correction analogous to that used by Akbari et al (lambda = original_lambda*(2.78/5.26)).

D bmws-admix simulations

To characterize the behavior of bmws-admix in different scenarios, we simulated populations over time with 3 different sampling strategies for a population with 3 admixture components:

1. 30 individuals every 5 generations (plus 100 inidividuals at present-day), with admixture proportions sampled by uniformly choosing 1 population resulting in the inclusion of unadmixed individuals.

2. 30 individuals every 5 generations (plus 100 inidividuals at present-day), with admixture proportions sampled from a uniform distribution, resulting in entirely admixed individuals.

3. Sampled to match the European sample sizes, timing, and admixture proportions.

We simulated selection coefficients ranging from $s = 0$ to $s = 0.1$ and varied the timing of the selection and whether it affected all three components or only one. In scenario 1, it can detect patterns affecting every component equally even down to $s = 0.02$ when selection is transient, or $s = 0.01$ when it is consistent (Supp. Fig. D1A-D). When selection only impacts one component this can still be detected down to quite small values of s (Supp. Fig. D1A-D), however in cases where the selected allele fixes, it struggles to capture the correct magnitude of selection for the whole time span (Supp. Fig. D1A-D).

In scenario 2, where every individual is admixed, while bmws-admix is again capable of detecting selection affecting all three components equally (Supp. Fig. D2A,B), it cannot detect selection when it impacts only one component (Supp. Fig. D2C,D). However it is not obvious how selection could affect only one component of ancestry in a real population where all individuals are admixed.

Finally, we therefore also simulated scenario 3, with the sampling and admixture in the European ancient data. Once again it could identify selection affecting all 3 components (Supp. Fig. D3A,B), but struggled when selection affects only 1 (Supp. Fig. D3C,D). As a general rule in the latter case, scenarios 2 and 3 had a narrow range of s that they could mostly detect, but tended to miss small values of s (< 0.05) entirely, and underestimate s when it approached 1.

Overall we find that bmws-admix works well when selection coefficients are the same across ancestries; in which case the different ancestral estimates for s should be regarded as distinct estimates of the same underlying parameters. It can also detect differing selection coefficients across ancestry as long as unadmixed individuals are included. It cannot detect different selection coefficients in different ancestries if all individuals are admixed. In particular, if selection and ancestry both vary with geography then the average selection coefficient experienced by each ancestry might differ; in this case bmws-admix will effectively estimate the average selection coefficient experienced by individuals in the sample.

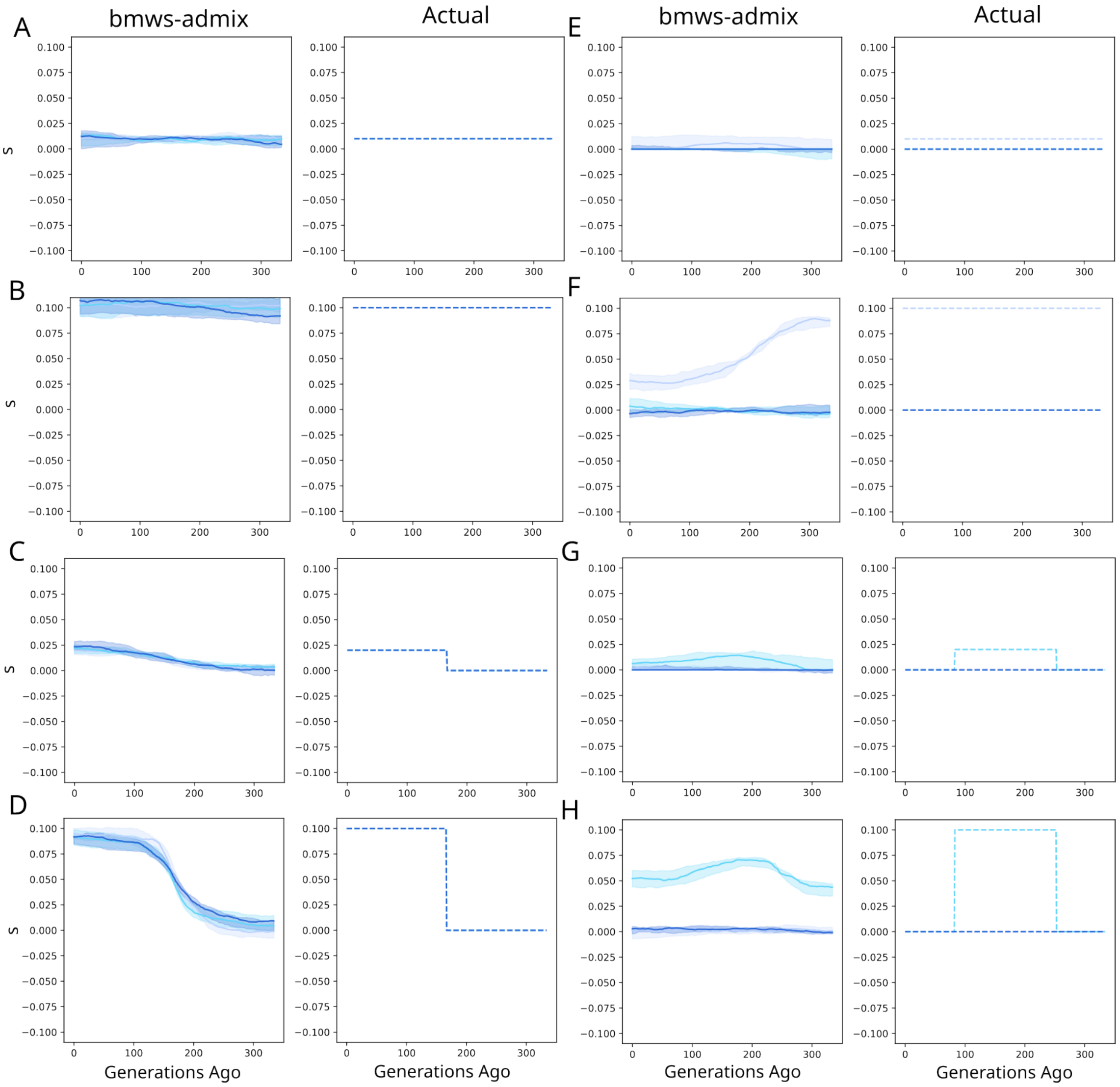

Supplementary Figure D1: Comparison of simulated selection and results from bmws-admix for simulations including unadmixed individuals

We simulated equal selection for all three components for A) $s = 0.01$ and B) $s = 0.1$ across the full time span, and C) $s = 0.02$ and D) $s = 0.1$ for the most recent half of the time. E-H) follow the same pattern, but only one component is under selection.

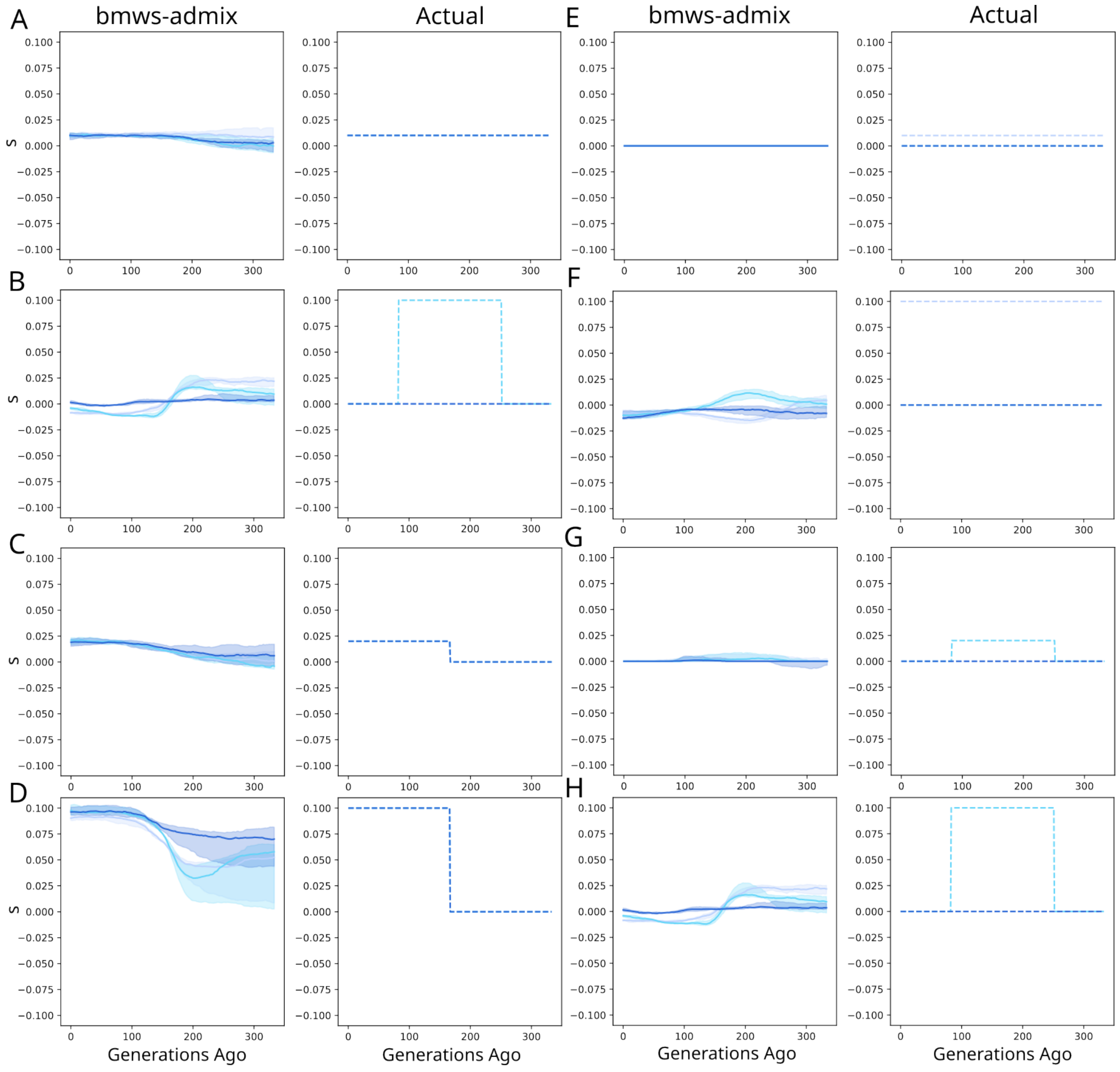

Supplementary Figure D2: Comparison of simulated selection and results from bmws-admix for simulations for entirely admixed individuals
We simulated equal selection for all three components for $s = 0.01$ across A) the full time span and B) the most recent half of the time, then selection only on one component at $s = 0.05$ C) the full time span and D) the middle half of the time.We simulated equal selection for all three components for A) $s = 0.01$ and B) $s = 0.1$ across the full time span, and C) $s = 0.02$ and D) $s = 0.1$ for the most recent half of the time. E-H) follow the same pattern, but only one component is under selection.

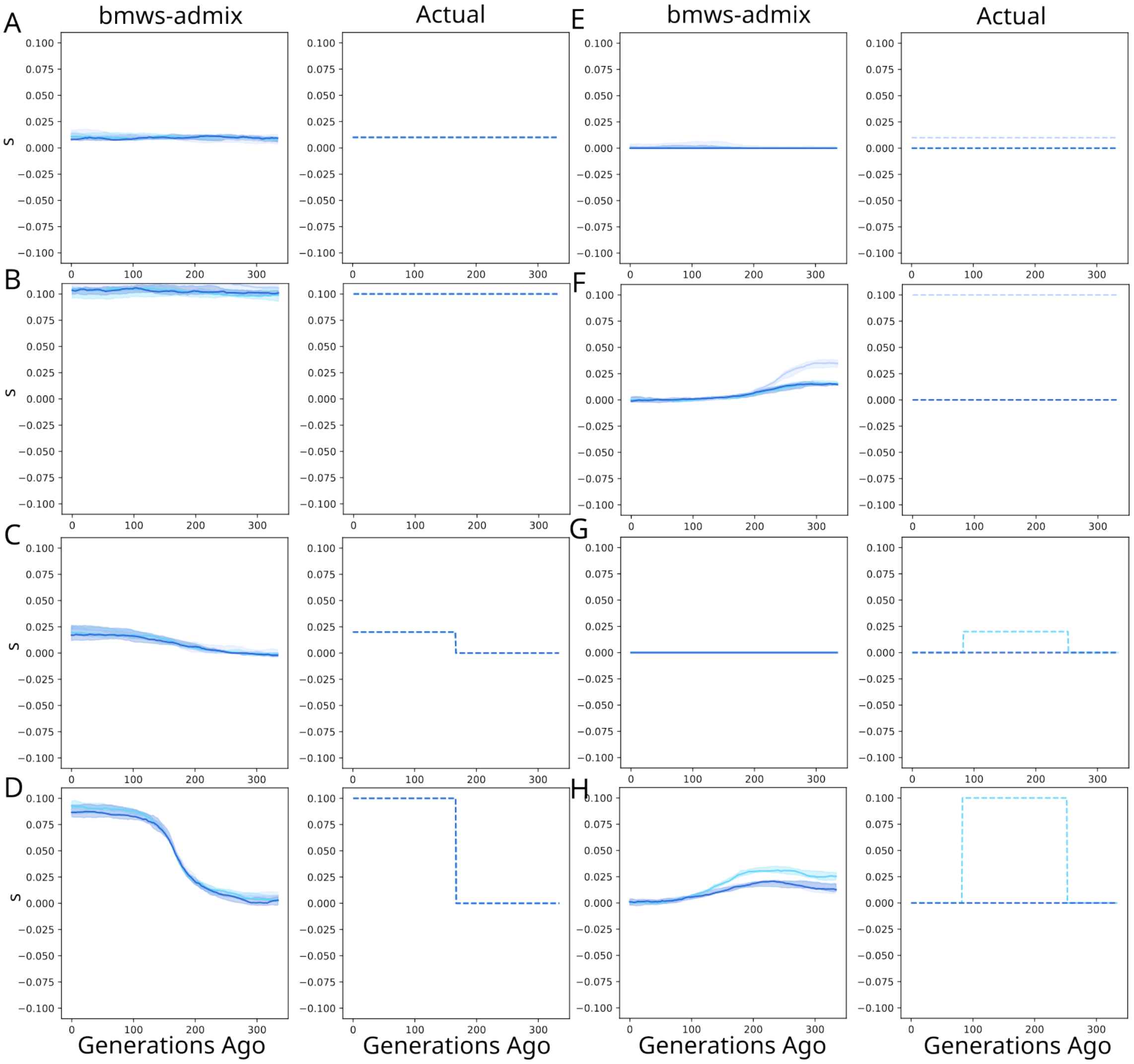

Supplementary Figure D3: Comparison of simulated selection and results from bmws-admix for simulations matching the timing, sampling, and admixture proportions of Europe

We simulated equal selection for all three components for A) $s = 0.01$ and B) $s = 0.1$ across the full time span, and C) $s = 0.02$ and D) $s = 0.1$ for the most recent half of the time. E-H) follow the same pattern, but only one component is under selection.

Supplementary Figures

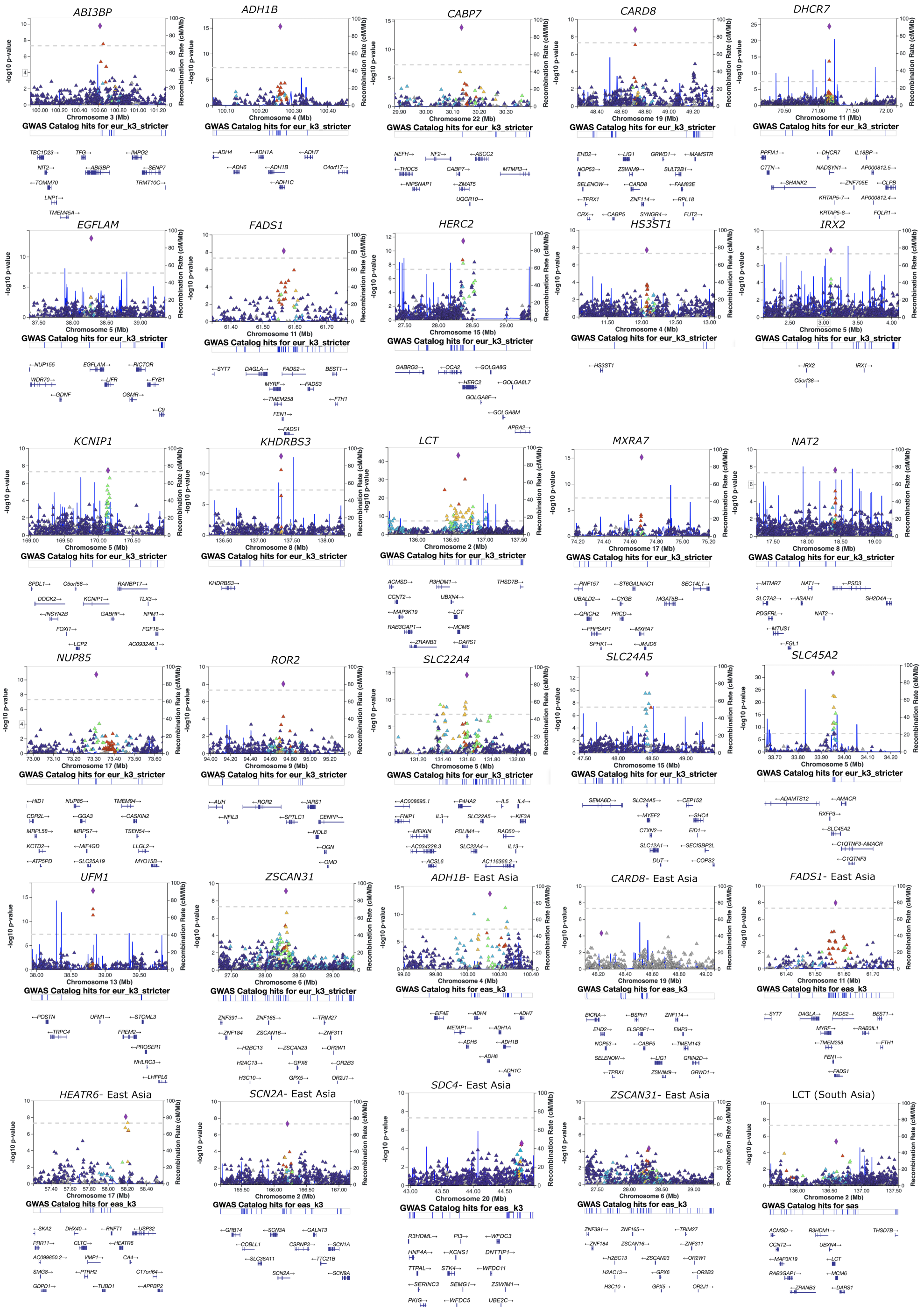

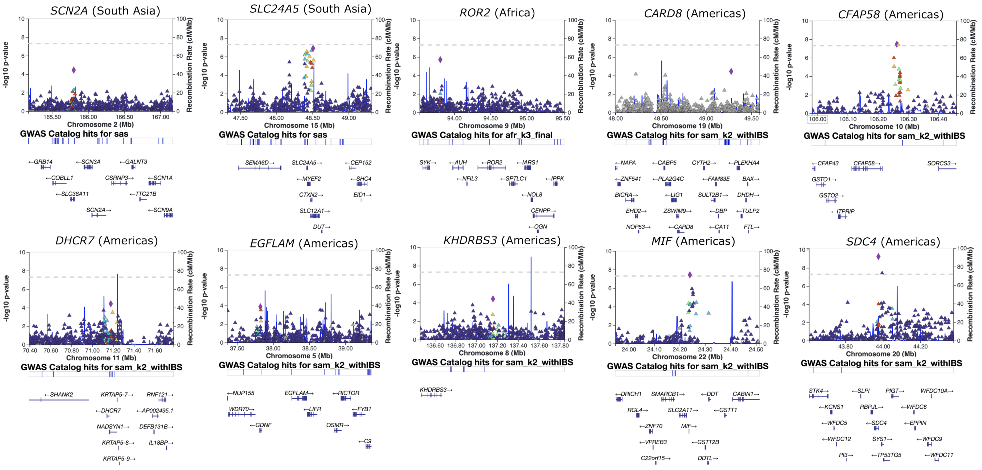

Supplementary Figure S1: Locus plots for significant and replicating peaks in all 5 geographic regions From left to right, we plotted Observed AFs, selection coefficient, and inferred AF trajectories. We plotted the information for the derived allele of the lead SNP in each peak. In the frequency plots, the error bars represent the 95% confidence intervals calculated using the Agresti-Coull method, while the red asterisk indicates the fitted frequency from the admixture model and the dotted line separates ancient (top) from present-day (bottom) groups. The pie charts indicate the average proportion of each admixture component for each population. Loci are ordered by region, then alphabetically by causal/nearest gene.

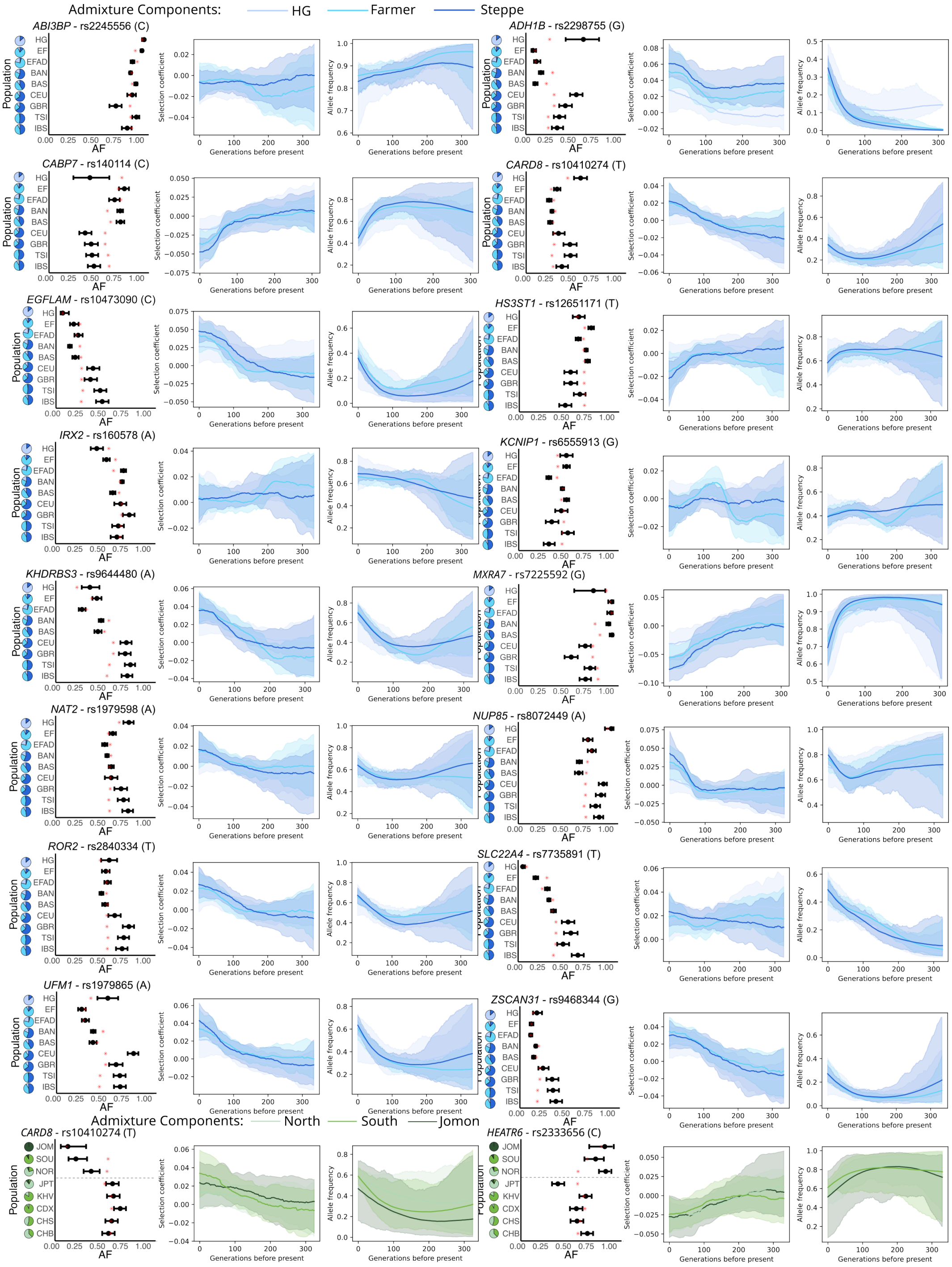
Admixture Components: HG Farmer Steppe
ABI3BP - rs2245556 (C)
ADH1B - rs2298755 (G)
CABP7 - rs140114 (C)
CARD8 - rs10410274 (T)
EGFLAM - rs10473090 (C)
HS3ST1 - rs12651171 (T)
IRX2 - rs160578 (A)
KCNIP1 - rs6555913 (G)
KHDRBS3 - rs9644480 (A)
MXRA7 - rs7225592 (G)
NAT2 - rs1979598 (A)
NUP85 - rs8072449 (A)
ROR2 - rs2840334 (T)
SLC22A4 - rs7735891 (T)
UFM1 - rs1979865 (A)
ZSCAN31 - rs9468344 (G)
Admixture Components: North South Jomon
CARD8 - rs10410274 (T)
HEATR6 - rs2333656 (C)
Population
AF
Selection coefficient
Allele frequency
Generations before present
HG EF EFAD BAN BAS CEU GBR TSI IBS
JOM SOU NOR JPT KHV CDX CHS CHB

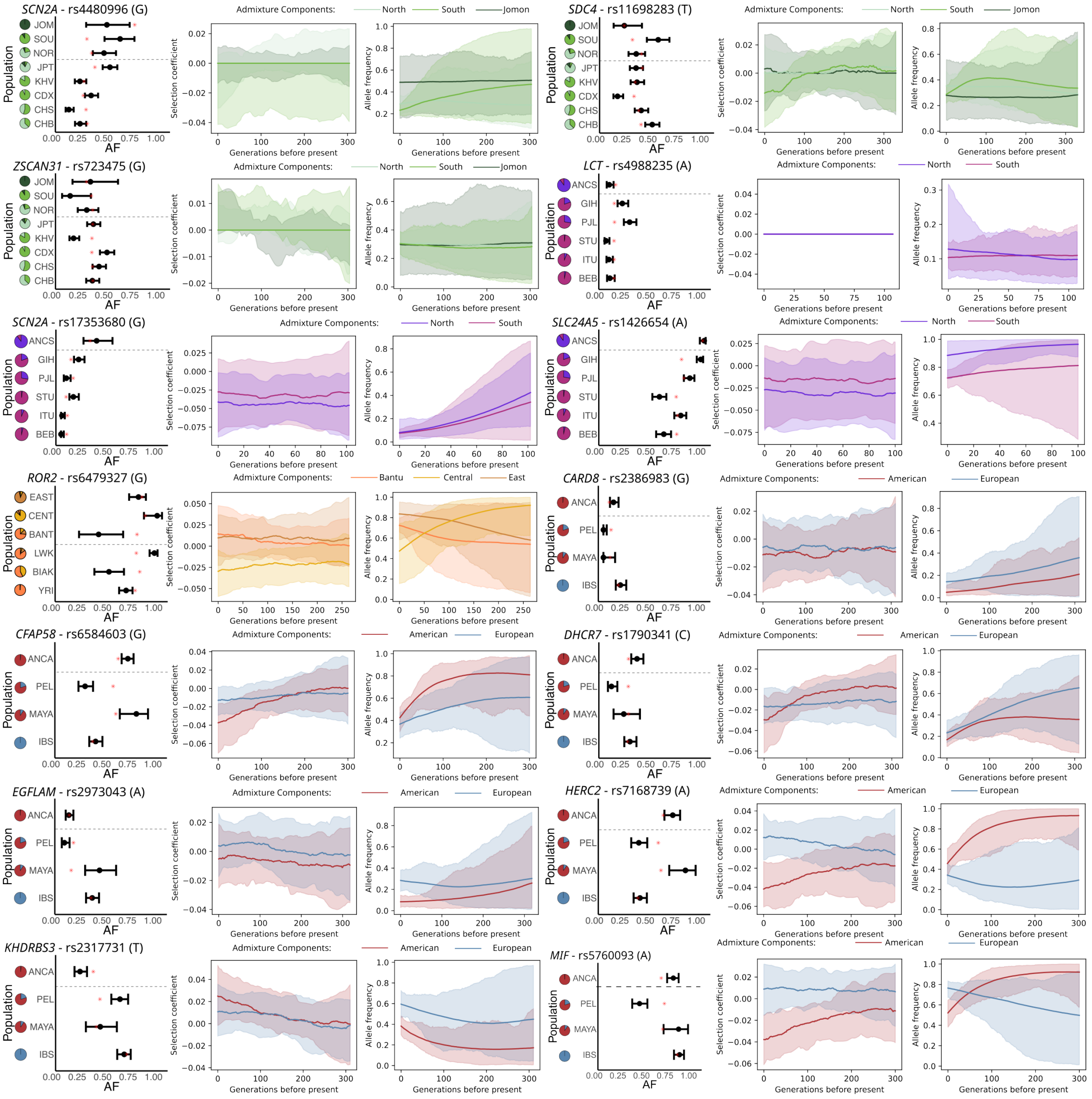

Supplementary Figure S2: Observed AF, Selection coefficient, and AF Trajectory plots for Bonferroni-significant and replication loci not highlighted in the main text.

Within each region, loci are ordered alphabetically by causal/nearest gene. We plotted the information for the derived allele of the lead SNP in each peak. In the frequency plots, the error bars represent the 95% confidence intervals calculated using the Agresti-Coull method, while the red asterisk indicates the fitted frequency from the admixture model and the dotted line separates ancient (top) from present-day (bottom) groups. The pie charts indicate the average proportion of each admixture component for each population.

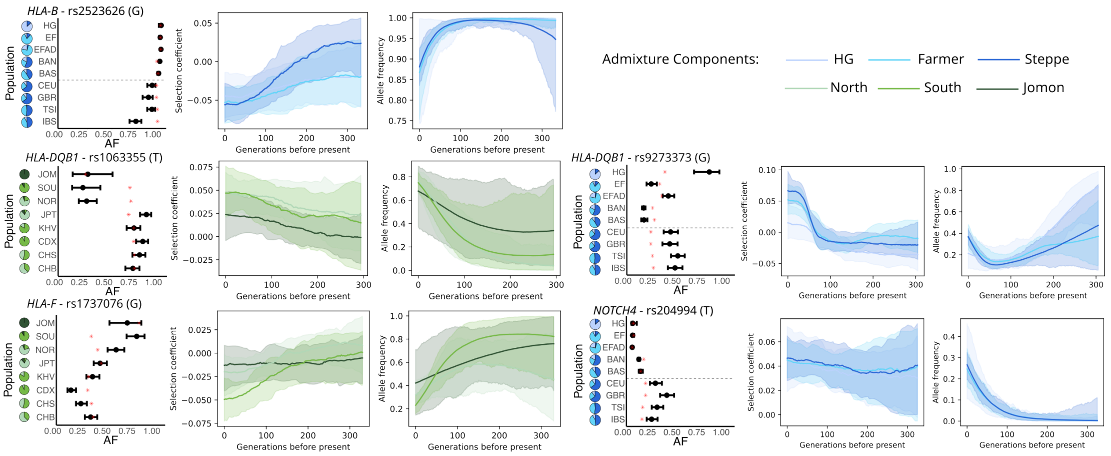

Supplementary Figure S3: Locus plots for significant and replicating peaks in the HLA region. From left to right, we plotted Observed AFs, selection coefficient, and inferred AF trajectories. We plotted the information for the derived allele of the lead SNP in each peak. In the frequency plots, the error bars represent the 95% confidence intervals calculated using the Agresti-Coull method, while the red asterisk indicates the fitted frequency from the admixture model and the dotted line separates ancient (top) from present-day (bottom) groups. The pie charts indicate the average proportion of each admixture component for each population.

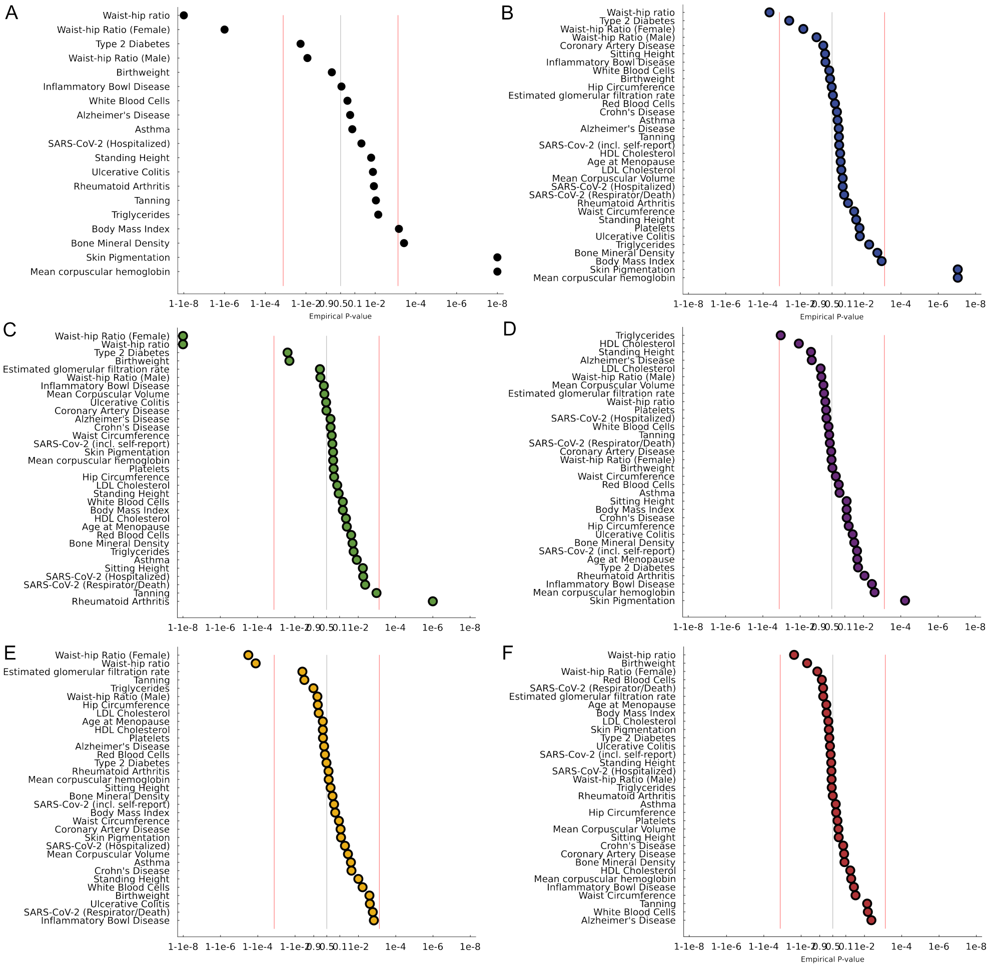

Supplementary Figure S4: Results of the polygenic tests run using European GWAS. A) Joint results for all 5 regions, and region-specific results for all 33 traits in B) Europe, C) East Asia, D) South Asia, E) Africa, and F) the Americas.

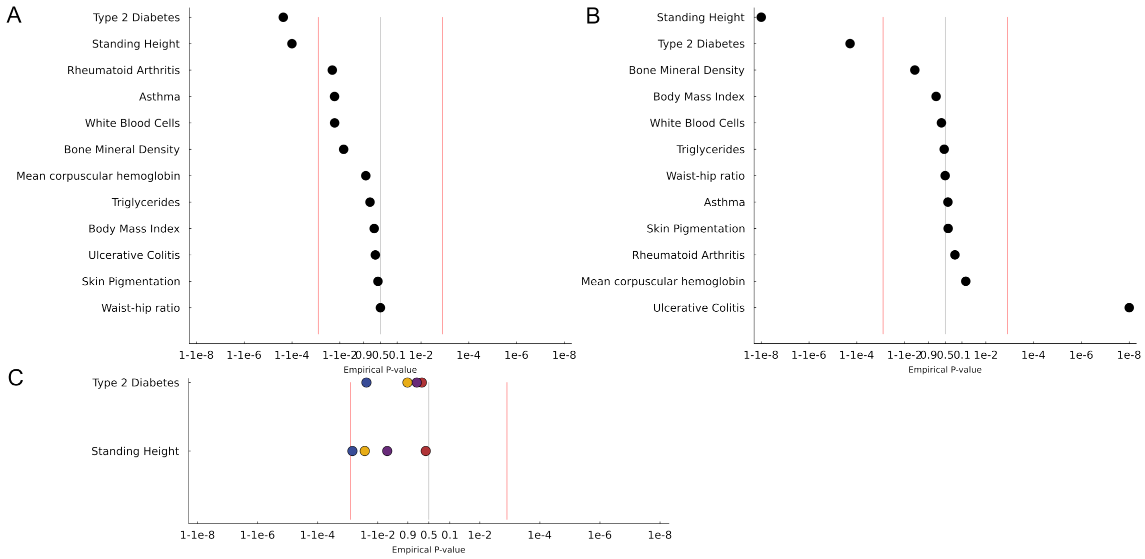

Supplementary Figure S5: Results of the joint polygenic test run using East Asian GWAS. A) A joint analysis without the East Asia region, which might be influenced by population stratification in the underlying GWAS, B) and the full joint analysis. C) Results from individual regions for those traits significant in the non-Stratified joint analysis.

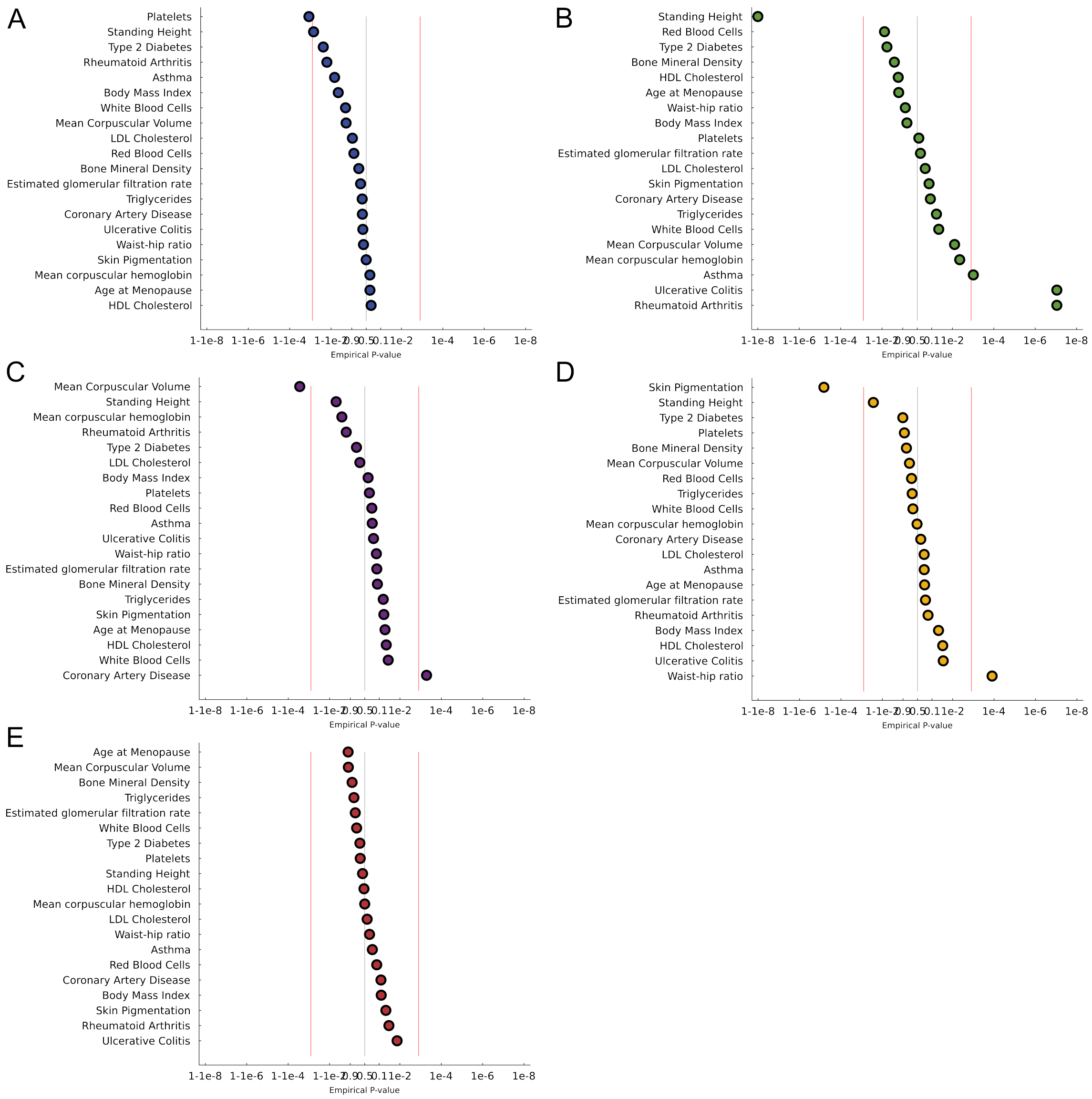

Supplementary Figure S6: Results of the polygenic tests run using East Asian GWAS.
Region-specific results for all 20 traits in A) Europe, B) East Asia, C) South Asia, D) Africa, and E) the Americas.

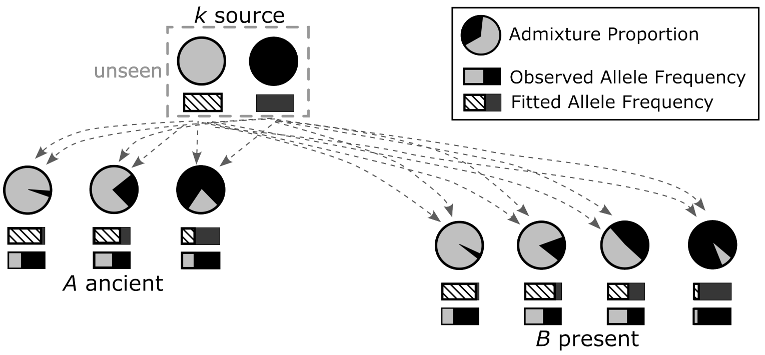

Supplementary Figure S7: We developed a test for selection by modeling the demographic history of populations.
A) We fit expected allele frequencies based on proportions of admixture of source populations in both ancient and modern populations (Methods). B) We tested for selection in four regions where we had data from both ancient and modern populations.

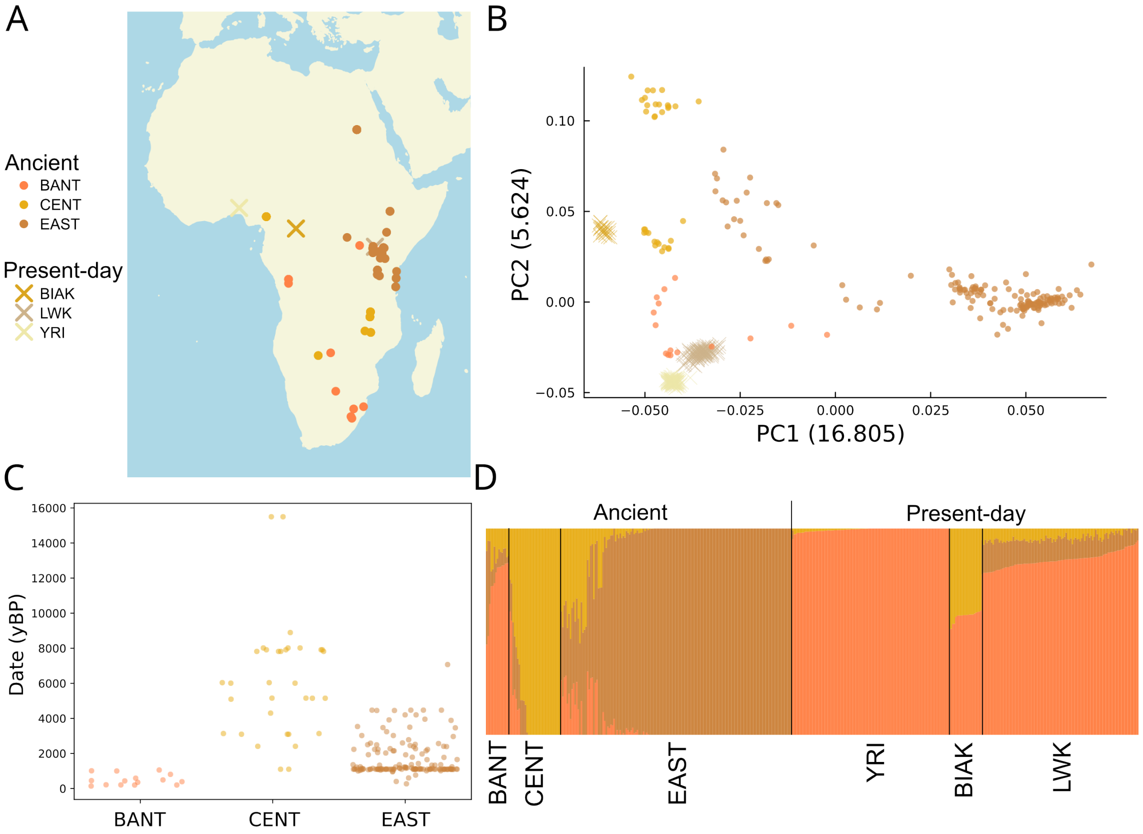

Supplementary Figure S8: Summary of the African groups used in this study.
A) Map of samples (for ancients) and collection points (for present-day). B) PCA with the 3 ancient and 3 present-day groups constructed using African samples from Human Origins dataset. C) Age distributions of ancient groups. D) ADMIXTURE plot of the ancient groups and present-day populations, run with $K = 3$. We took the mean (weighted by coverage) of these admixture proportions for each population to use for the selections scans. BANT= Bantu; CENT = Central African; EAST = East African; YRI = Yoruba, LWK = Luhya, BIAK = Biaka.

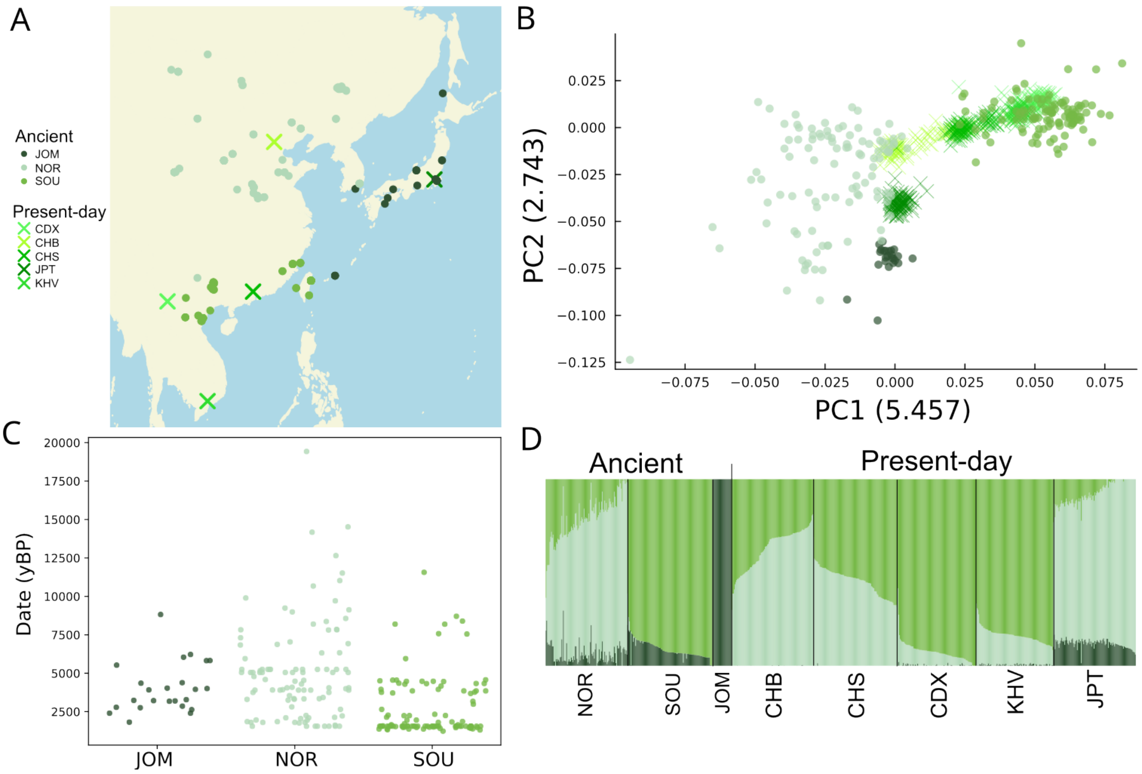

Supplementary Figure S9: Summary of the East Asian groups used in this study.
A) Map of samples (for ancients) and collection points (for present-day). B) PCA with the 3 ancient and 5 present-day groups constructed using Asian samples from Human Origins dataset. C) Age distributions of ancient groups. D) ADMIXTURE plot of the ancient groups and present-day populations, run with $K = 3$. We took the mean (weighted by coverage) of these admixture proportions for each population to use for the selections scans. NOR = Northern Mainland; SOU = Southern Mainland; JOM = Jomon; CHB = Han Chinese (Beijing); CHS = Han Chinese (South); CDX = Dai; KHV = Vietnamese; JPT = Japanese.

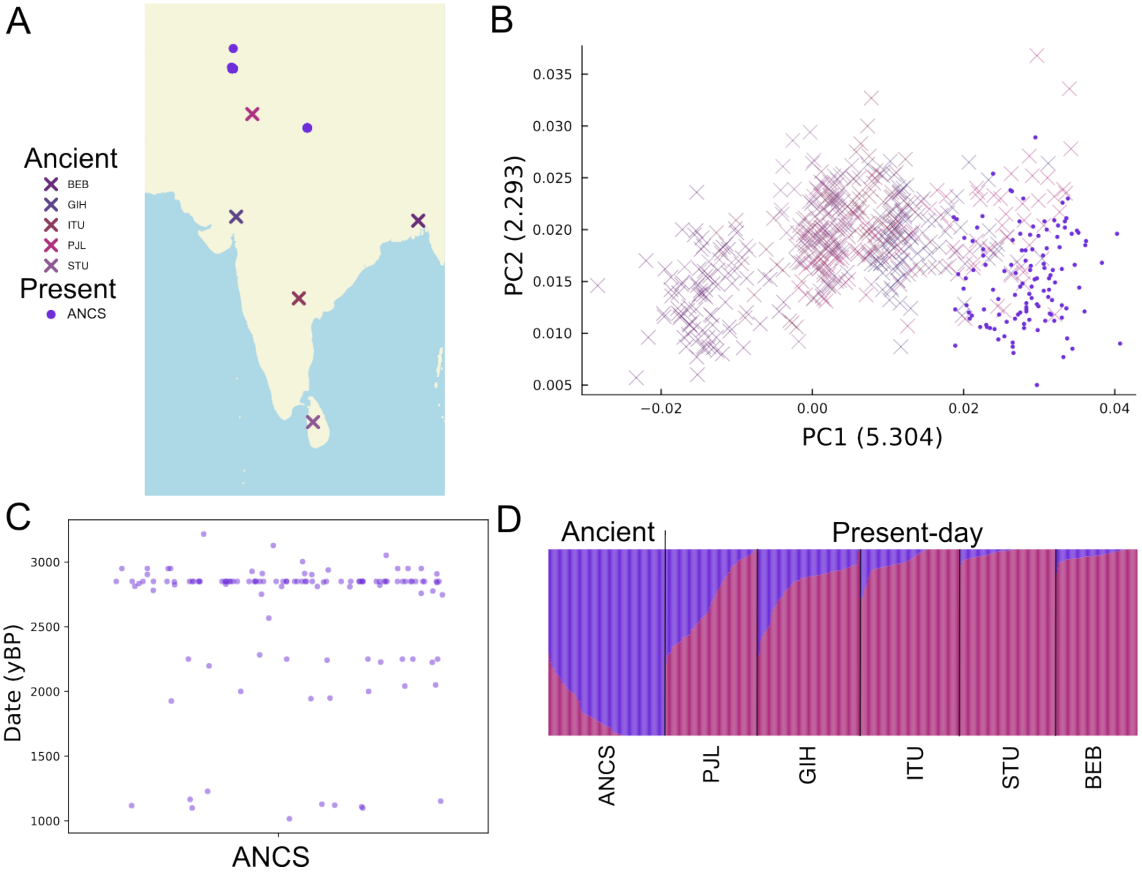

Supplementary Figure S10: Summary of the South Asian groups used in this study.
A) Map of samples (for ancients) and collection points (for present-day). B) PCA with the 1 ancient and 5 present-day groups constructed South Asian samples from the Human Origins dataset. C) Age distributions of ancient group. D) ADMIXTURE plot of the ancient groups and present-day populations, run with $K = 2$. We took the mean (weighted by coverage) of these admixture proportions for each population to use for the selections scans. ANCS = Ancient; PJL = Punjabi; GIH = Gujarati; ITU = Telugu; STU = Tamil; BEB = Bengali.

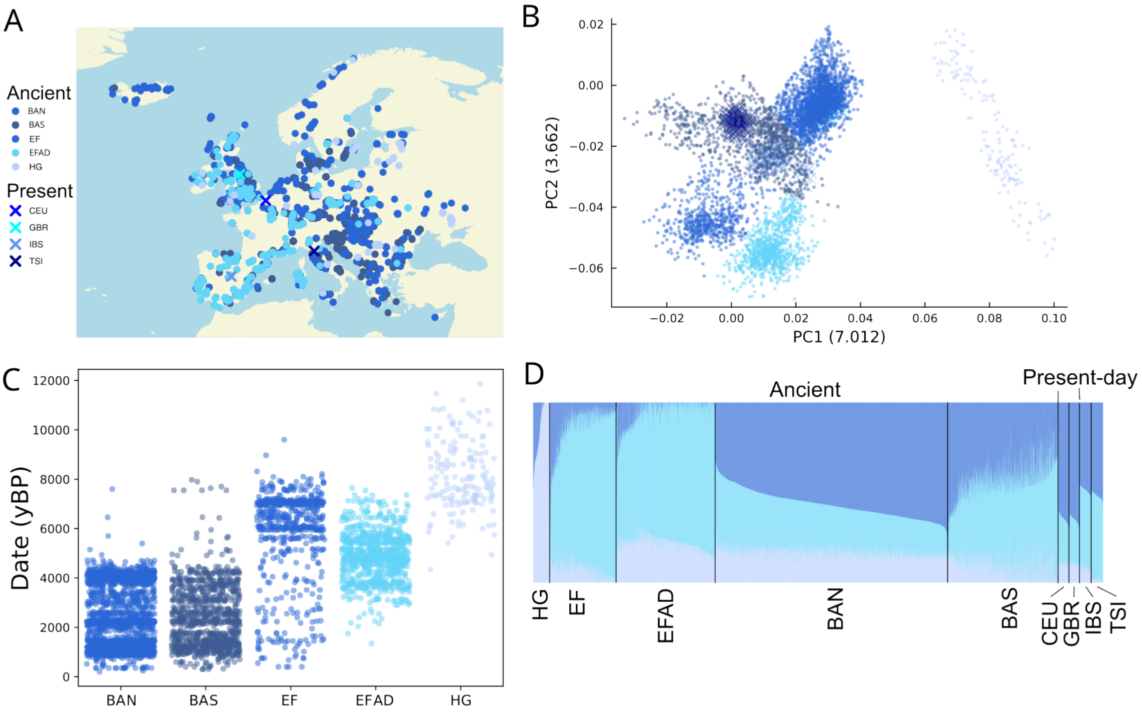

Supplementary Figure S11: Summary of the European groups used in this study.
A) Map of samples (for ancients) and collection points (for present-day). B) PCA with the 5 ancient and 4 present-day groups constructed using Non-Finnish European samples from Human Origins dataset. C) Age distributions of ancient groups. D) ADMIXTURE plot of the ancient groups and present-day populations, run with $K = 3$. We took the mean (weighted by coverage) of these admixture proportions for each population to use for the selection scans. HG = Hunter-gatherer; EF = Early Farmer; EFAD = Early Farmer (Admixed); BAN = Bronze Age (North); BAS = Bronze Age (South); CEU = CEPH Northwestern European; GBR = British; IBS = Spanish; TSI = Tuscan.

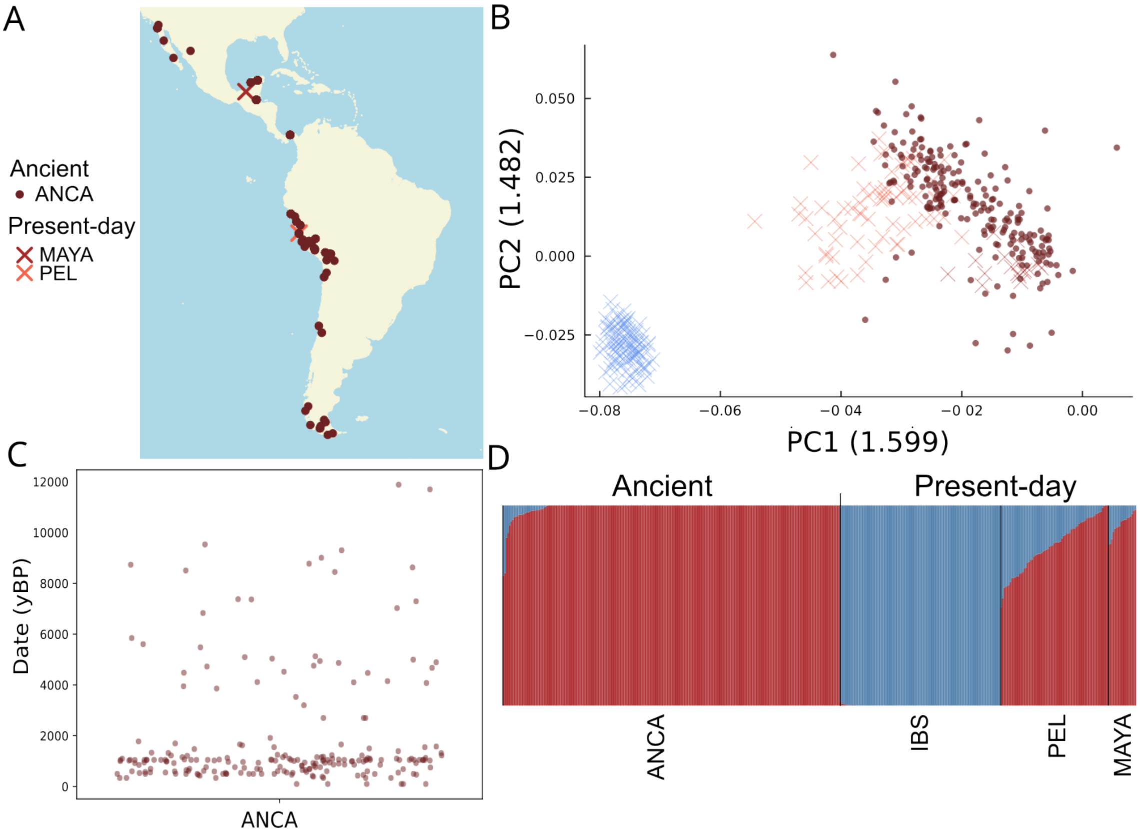

Supplementary Figure S12: Summary of the American groups used in this study.
A) Map of samples (for ancients) and collection points (for present-day; excluding IBS, which is marked on the Europe map in Supp. Fig. S11. B) PCA with the 1 ancient and 3 present-day groups constructed using Asian samples from Human Origins dataset. C) Age distributions of ancient groups. D) ADMIXTURE plot of the ancient groups and present-day populations, run with $K = 2$. We took the mean (weighted by coverage) of these admixture proportions for each population to use for the selections scans. ANCA = Ancient; IBS = Spanish; PEL = Peruvian; MAYA = Mayan.

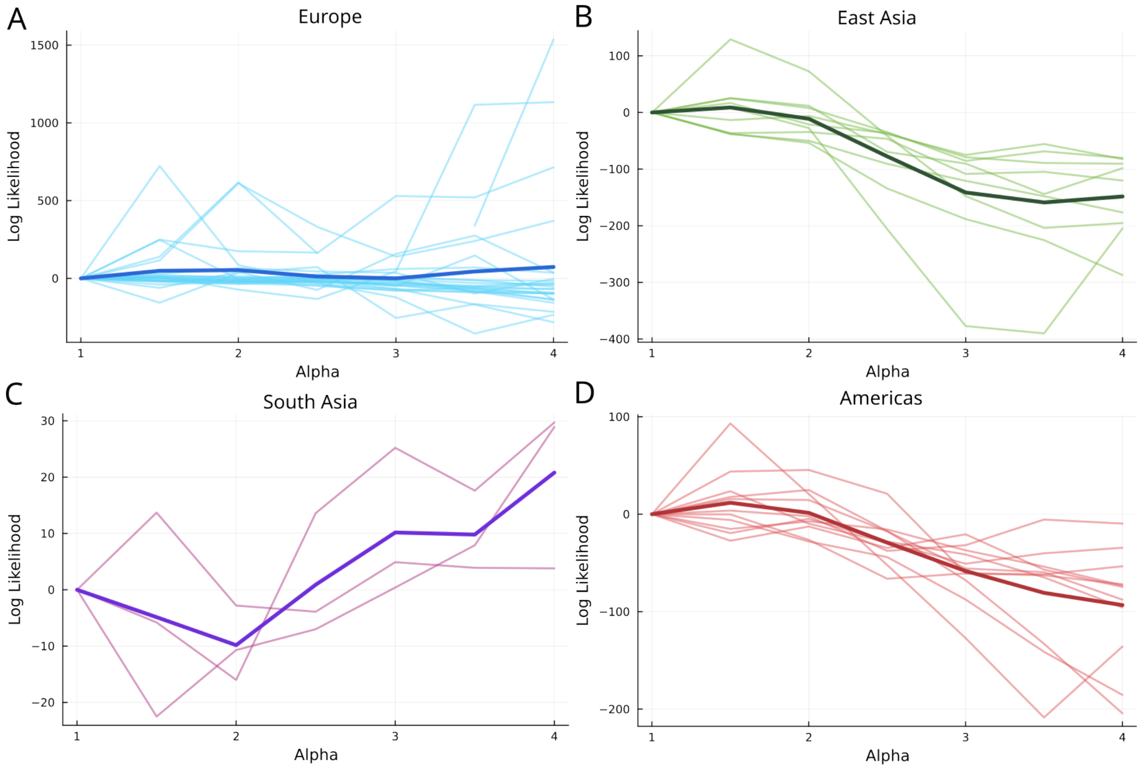

Supplementary Figure S13: Log Likelihoods for the time series selection scan plotted against a range of smoothing parameters for A) Europe, B) East Asia, C) South Asia, and D) the Americas. Each lighter line is a variant, while the dark one is the average of all variants. For each line the likelihood is transformed by adding the likelihood at $\alpha = 1$, such that each starts at zero. Based on this, we used $\alpha = 2$ for all analyses in the paper.

Supplementary Tables

Locus	Variant(s)	Novelty
SLC24A5	Europe (South Asia)	Known
LCT	Europe (South Asia)	Known
HERC2	Europe (Americas)	Known
SLC45A2	Europe	Known
SLC22A4	Europe	Known
DHCR7	Europe (Americas)	Known
ADH1B	East Asia (Europe)	Known
FADS1	East Asia (Europe)	Known
HEATR6	East Asia	novel
SCN2A	East Asia (South Asia)	novel
IRX2	Europe	novel
KCNIP1	Europe	novel
ABI3BP	Europe	novel
MXRA7	Europe	novel
CARD8	Europe (East Asia, Americas)	novel
EGFLAM	Europe (Americas)	novel
HS3ST1	Europe	novel
CABP7	Europe	novel
NAT2	Europe	novel
UFM1	Europe	novel
ROR2	Europe (Africa)	novel
KHDRBS3	Europe (Americas)	novel
NUP85	Europe	novel
ZSCAN31	Europe (East Asia)	novel
SDC4	Americas (East Asia)	novel
MIF	Americas	novel
CFAP58	Americas	novel
HLA-DQB1	Europe (East Asia)	Known
HLA-B	Europe (Americas)	Known
HLA-F	Americas (East Asia)	Known

Supplementary Table 1: Table of all loci passing a Bonferroni multiple testing correction in at least on region. Regions in parentheses indicate those that at least nominally replicate the peak (Methods).

Trait	SNPs	AFR. P	EAS P	EUR P	SAS P	AMR P	Joint	NonEas
Asthma	64	0.238	0.001	0.985	0.219	0.213	0.387	0.994
Bone Mineral Density	190	0.855	0.961	0.783	0.123	0.877	0.973	0.986
Body Mass Index	264	0.048	0.841	0.978	0.346	0.080	0.793	0.726
Coronary Artery Disease	79	0.348	0.116	0.675	0.001	0.082	0.150	0.595
Est. glomerular filtration rate	61	0.207	0.348	0.735	0.132	0.825	0.604	0.936
HDL Cholesterol	401	0.030	0.939	0.292	0.046	0.539	0.389	0.314
Standing Height	1762	0.996	1.000	0.999	0.979	0.605	1.000	1.000
LDL Cholesterol	212	0.239	0.205	0.893	0.711	0.381	0.701	0.936
Triglycerides	343	0.724	0.059	0.682	0.064	0.849	0.548	0.817
Mean corpuscular hemoglobin	324	0.526	0.004	0.335	0.961	0.498	0.069	0.878
Mean corpuscular volume	330	0.796	0.008	0.947	1.000	0.919	0.398	1.000
Age at Menopause	45	0.228	0.936	0.332	0.053	0.921	0.848	0.715
Platelet Counts	875	0.884	0.418	0.999	0.301	0.695	0.989	1.000
Rheumatoid Arthritis	248	0.155	0.00	0.994	0.936	0.034	0.197	0.995
Red Blood Cell Count	453	0.743	0.987	0.874	0.228	0.131	0.966	0.895
Skin Pigmentation	18	1.000	0.134	0.505	0.060	0.047	0.378	0.607
Type II Diabetes	353	0.903	0.983	0.996	0.802	0.707	1.000	1.000
Ulcerative Colitis	59	0.029	0.00	0.659	0.190	0.014	1E-8	0.694
White Blood Cell Counts	360	0.700	0.046	0.950	0.037	0.796	0.653	0.994
Waist-Hip Ratio	51	1E-4	0.867	0.633	0.138	0.292	0.504	0.501

Supplementary Table 2: Results of testing for polygenic selection on 19 traits with East Asian-ancestry GWAS. Fields include the trait abbreviation, short description, number of variants included in the test, and the p-values in Africa, East Asia, Europe, and the Americas. Loci for all traits were those that passed a $p < 1e-8$ threshold in the original GWAS except for † traits, where we used $1e-6$ instead. *passes Bonferroni correction < 0.05.

Trait	SNPs	AFR. P	EAS P	EUR P	SAS P	AMR P	Joint	NonEur
Alzheimer's Disease	42	0.640	0.307	0.219	0.958	0.004	0.170	0.192
Asthma	586	0.025	0.012	0.251	0.193	0.348	0.133	0.014
Bone Mineral Density	1080	0.246	0.020	0.002	0.031	0.118	3.84E-4	0.112
Body Mass Index	831	0.176	0.067	0.001	0.081	0.768	0.001	0.704
Birthweight	115	0.003	0.995	0.608	0.466	0.979	0.814	0.918
Crohn's Disease	263	0.024	0.304	0.272	0.080	0.139	0.184	0.338
Covid-19 (Respirator/Death)	69	0.002	0.004	0.111	0.610	0.849	0.037	0.030
Covid-19 (Hospitalized)	70	0.053	0.005	0.133	0.743	0.576	0.047	0.027
Covid-19 (incl. self-report)	43	0.203	0.244	0.207	0.022	0.617	0.179	0.585
Coronary Artery Disease	391	0.091	0.507	0.831	0.535	0.124	0.754	0.409
Est. glomerular filtration rate	281	0.975	0.779	0.449	0.809	0.847	0.622	0.992
HDL Cholesterol	1238	0.693	0.046	0.183	0.991	0.058	0.117	0.155
Standing Height	4102	0.010	0.112	0.025	0.962	0.587	0.016	0.173
Hip Circumference	810	0.835	0.196	0.517	0.062	0.331	0.481	0.681
Inflammatory Bowl Disease	310	0.001	0.641	0.778	0.004	0.037	0.454	0.238
LDL Cholesterol	1493	0.815	0.133	0.158	0.875	0.712	0.276	0.811
Triglycerides	1371	0.904	0.018	0.005	0.999	0.562	0.007	0.620
Mean corpuscular hemoglobin	478	0.395	0.221	0.000	0.003	0.052	1E-8	0.331
Mean corpuscular volume	1846	0.037	0.618	0.134	0.822	0.250	0.211	0.575
Age at Menopause	143	0.694	0.041	0.172	0.022	0.775	0.067	0.461
Platelet Counts	2160	0.687	0.209	0.017	0.753	0.281	0.022	0.680
Rheumatoid Arthritis	621	0.404	1E-6	0.069	0.009	0.502	0.012	1.5E-5
Red Blood Cell Count	1451	0.600	0.024	0.345	0.212	0.871	0.207	0.263
Sitting Height	2640	0.313	0.006	0.787	0.081	0.241	0.205	0.053
Skin Pigmentation	657	0.086	0.224	0.000	5.9E-5	0.694	1E-8	0.807
Type II Diabetes	423	0.518	0.996	0.997	0.020	0.674	0.995	1.000
Tanning	654	0.969	0.001	0.213	0.638	0.007	0.009	0.002
Ulcerative Colitis	202	0.002	0.527	0.016	0.038	0.647	0.013	0.384
Waist Circumference	497	0.112	0.264	0.032	0.305	0.030	0.016	0.242
White Blood Cell Counts	1372	0.006	0.067	0.646	0.668	0.007	0.231	0.010
Waist-Hip Ratio	1362	1.000	1.000	1.000	0.776	0.996	1.000	1.000
Waist-Hip Ratio (Females)	978	1.000	1.000	0.986	0.518	0.926	1.000	1.000
Waist-Hip Ratio (Males)	338	0.843	0.766	0.926	0.863	0.569	0.988	0.971

Supplementary Table 3: Results of testing for polygenic selection on 33 traits with European-ancestry GWAS. Fields include the trait, number of variants included in the test, and the p-values in Africa, East Asia, South Asia, Europe, and the Americas, as well as the joint analysis. Loci for all traits were those that passed a $p < 1e - 8$ threshold in the original GWAS. *passes two-sided Bonferroni correction < 0.05.

References

[1] Iain Mathieson, Iosif Lazaridis, Nadin Rohland, et al. Genome-wide patterns of selection in 230 ancient eurasians. *Nature*, 528:499–503, 2015.

[2] Ali Akbari, Alison R Barton, Steven Gazal, et al. Pervasive findings of directional selection realize the promise of ancient dna to elucidate human adaptation. *bioRxiv*, page 2024.09.14.613021, 2024.

[3] Evan K Irving-Pease, Alba Refoyo-Martínez, William Barrie, et al. The selection landscape and genetic legacy of ancient eurasians. *Nature*, 625:312–320, 2024.

[4] Gaspard Kerner, Anna-Lena Neehus, Quentin Philippot, et al. Genetic adaptation to pathogens and increased risk of inflammatory disorders in post-Neolithic Europe. *Cell genomics*, 3(2):100248, 2023.

[5] Kazuya Okamoto, Masaki Ohashi, Kana Ohno, et al. Involvement of NIPSNAP1, a neuropeptide nocistatin-interacting protein, in inflammatory pain. *Molecular pain*, 12, 2016.

[6] Soh Yamamoto, Noriko Ogasawara, Keisuke Yamamoto, et al. Mitochondrial proteins NIP-SNAP-1 and -2 are a target for the immunomodulatory activity of clarithromycin, which involves NF-κB-mediated cytokine production. *Biochemical and biophysical research communications*, 483(3): 911–916, 2017.

[7] Elliot Sollis, Abayomi Mosaku, Ala Abid, et al. The NHGRI-EBI GWAS Catalog: knowledgebase and deposition resource. *Nucleic acids research*, 51(D1):D977–D985, 2023.

[8] GTEx Consortium. Genetic effects on gene expression across human tissues. *Nature*, 550:204–213, 2017.

[9] Michael R Knowles, Margaret W Leigh, Lawrence E Ostrowski, et al. Exome sequencing identifies mutations in CCDC114 as a cause of primary ciliary dyskinesia. *American journal of human genetics*, 92(1):99–106, 2013.

[10] Alexandros Onoufriadis, Tamara Paff, Dinu Antony, et al. Splice-site mutations in the axonemal outer dynein arm docking complex gene CCDC114 cause primary ciliary dyskinesia. *American journal of human genetics*, 92(1):88–98, 2013. doi: 10.1016/j.ajhg.2012.11.002.

[11] Jessie Kulsuptrakul, Elizabeth A Turcotte, Michael Emerman, and Patrick S Mitchell. A human-specific motif facilitates CARD8 inflammasome activation after HIV-1 infection. *eLife*, 12, 2023.

[12] Tugay Karakaya, Marta Slaufova, Michela Di Filippo, et al. CARD8: A Novel Inflammasome Sensor with Well-Known Anti-Inflammatory and Anti-Apoptotic Activity. *Cells*, 13(12), 2024. doi: 10.3390/cells13121032.

[13] Zheng-Zheng Li, Wen-Long Zhao, Gui-Shuan Wang, et al. The novel testicular enrichment protein Cfap58 is required for Notch-associated ciliogenesis. *Bioscience reports*, 40(1), 2020.

[14] Yibin Xu, Armand 3rd Bankhead, Xiaoli Tian, et al. Deletion of Glutathione S-Transferase Omega 1 Activates Type I Interferon Genes and Downregulates Tissue Factor. *Cancer research*, 80(17): 3692–3705, 2020.

[15] D M Escobar-García, L M Del Razo, L C Sanchez-Peña, et al. Association of glutathione S-transferase Ω 1-1 polymorphisms (A140D and E208K) with the expression of interleukin-8 (IL-8), transforming growth factor beta (TGF-β), and apoptotic protease-activating factor 1 (Apaf-1) in

humans chronically exposed to arsenic in drinking water. *Archives of Toxicology*, 86(6):857–868, 2012.

[16] Lanrong Luo, Yuanyuan Li, Yanhui Gao, et al. Association between arsenic metabolism gene polymorphisms and arsenic-induced skin lesions in individuals exposed to high-dose inorganic arsenic in northwest China. *Scientific reports*, 8(1):413, 2018.

[17] Moumita Majumder, Uma B Dasgupta, D N Guha Mazumder, and Nilansu Das. Skin score correlates with global DNA methylation and GSTO1 A140D polymorphism in arsenic-affected population of Eastern India. *Toxicology mechanisms and methods*, 27(6):467–475, 2017.

[18] Deborah Sterling and Joseph R Casey. Bicarbonate transport proteins. *Biochemistry and Cell Biology*, 80(5):483–497, 2002.

[19] Michael Klier, Fabian T Andes, Joachim W Deitmer, and Holger M Becker. Intracellular and Extracellular Carbonic Anhydrases Cooperate Non-enzymatically to Enhance Activity of Monocarboxylate Transporters*. *Journal of Biological Chemistry*, 289(5):2765–2775, 2014.

[20] Linda S Forero-Quintero, Samantha Ames, Hans-Peter Schneider, et al. Membrane-anchored carbonic anhydrase IV interacts with monocarboxylate transporters via their chaperones CD147 and GP70. *Journal of Biological Chemistry*, 294(2):593–607, 2019.

[21] Nicholas W Shworak, Jian Liu, Linda M S Fritze, et al. Molecular Cloning and Expression of Mouse and Human cDNAs Encoding Heparan Sulfate d-Glucosaminyl 3-O-Sulfotransferase*. *Journal of Biological Chemistry*, 272(44):28008–28019, 1997.

[22] Yao Xu, Weilin Shi, Ruhui Song, et al. Divergent patterns of genic copy number variation in KCNIP1 gene reveal risk locus of type 2 diabetes in Chinese population. *Endocrine journal*, 65(5):537–545, 2018.

[23] Heun-Sik Lee, Sanghoon Moon, Jun Ho Yun, et al. Genome-wide copy number variation study reveals KCNIP1 as a modulator of insulin secretion. *Genomics*, 104(2):113–120, 2014.

[24] W Y Weiser, P A Temple, J S Witek-Giannotti, et al. Molecular cloning of a cDNA encoding a human macrophage migration inhibitory factor. *Proceedings of the National Academy of Sciences of the United States of America*, 86(19):7522–7526, 1989.

[25] Zhenjiang Sun, Peng Ke, Ying Shen, et al. MXRA7 is involved in monocyte-to-macrophage differentiation. *Molecular immunology*, 171:12–21, 2024.

[26] Francesca Luca, Giuseppina Bubba, Massimo Basile, et al. Multiple advantageous amino acid variants in the NAT2 gene in human populations. *PloS one*, 3(9):e3136, 2008.

[27] Hélène Magalon, Etienne Patin, Frédéric Austerlitz, et al. Population genetic diversity of the NAT2 gene supports a role of acetylation in human adaptation to farming in Central Asia. *European journal of human genetics : EJHG*, 16(2):243–251, 2008.

[28] Laura L Colbran, Maya R Johnson, Iain Mathieson, and John A Capra. Tracing the Evolution of Human Gene Regulation and Its Association with Shifts in Environment. *Genome Biology and Evolution*, 13(11):evab237, 2021.

[29] M Blum, D M Grant, W McBride, et al. Human arylamine N-acetyltransferase genes: isolation, chromosomal localization, and functional expression. *DNA and cell biology*, 9(3):193–203, 1990.

[30] Etsuko Toda, Yuya Terashima, Tsuyoshi Sato, et al. FROUNT Is a Common Regulator of CCR2 and CCR5 Signaling to Control Directional Migration. *The Journal of Immunology*, 183(10):6387–6394, 2009.

[31] Abraham L. Brass, Derek M. Dykxhoorn, Yair Benita, et al. Identification of Host Proteins Required for HIV Infection Through a Functional Genomic Screen. *Science*, 319(5865):921–926, 2008.

[32] Yue-Huan Ling, Hao Wang, Mei-Qing Han, et al. Nucleoporin 85 interacts with influenza A virus PB1 and PB2 to promote its replication by facilitating nuclear import of ribonucleoprotein. *Frontiers in Microbiology*, 13:895779, 2022.

[33] Lin Poyraz, Laura L Colbran, and Iain Mathieson. Predicting functional consequences of recent natural selection in britain. *Molecular Biology and Evolution*, 41:msae053, 2024.

[34] Dan Lv, Yang Luo, Wei Yang, et al. A novel single-base deletion in ROR2 causes atypical brachydactyly type B1 with cutaneous syndactyly in a large Chinese family. *Journal of human genetics*, 54(7):422–425, 2009.

[35] B Weiss and W Stoffel. Human and murine serine-palmitoyl-CoA transferase–cloning, expression and characterization of the key enzyme in sphingolipid synthesis. *European journal of biochemistry*, 249(1):239–247, 1997.

[36] Carina M Schlebusch, Pontus Skoglund, Per Sjödin, et al. Genomic Variation in Seven Khoe-San Groups Reveals Adaptation and Complex African History. *Science*, 338(6105):374 LP – 379, 2012.

[37] Junkyung Kwak and Dayeon Shin. Gene-Nutrient Interactions in Obesity: COBLL1 Genetic Variants Interact with Dietary Fat Intake to Modulate the Incidence of Obesity. *International journal of molecular sciences*, 24(4), 2023.

[38] Viktoria Glunk, Samantha Laber, Nasa Sinnott-Armstrong, et al. A non-coding variant linked to metabolic obesity with normal weight affects actin remodelling in subcutaneous adipocytes. *Nature metabolism*, 5(5):861–879, 2023.

[39] Zélia Ferreira, Belen Hurle, Jorge Rocha, and Susana Seixas. Differing evolutionary histories of WFDC8 (short-term balancing) in Europeans and SPINT4 (incomplete selective sweep) in Africans. *Molecular biology and evolution*, 28(10):2811–2822, 2011.

[40] Zélia Ferreira, Susana Seixas, Aida M Andrés, et al. Reproduction and immunity-driven natural selection in the human WFDC locus. *Molecular biology and evolution*, 30(4):938–950, 2013.

[41] Zélia Ferreira, Belen Hurle, Aida M Andrés, et al. Sequence diversity of Pan troglodytes subspecies and the impact of WFDC6 selective constraints in reproductive immunity. *Genome biology and evolution*, 5(12):2512–2523, 2013.

[42] C. D. Huff, D. J. Witherspoon, Y. Zhang, et al. Crohn's disease and genetic hitchhiking at ibd5. *Mol Biol Evol*, 29(1):101–11, 2012.

[43] Sayanika Banerjee, Manoj Kumar, and Reuven Wiener. Decrypting UFMylation: How Proteins Are Modified with UFM1. *Biomolecules*, 10(10), 2020.
